# In Situ Transmission Electron Microscopy Observation of Redox–Structure-Coupled Resistive Switching in High-Entropy Oxide Memristors

**DOI:** 10.1021/acsnano.6c09072

**Published:** 2026-08-26

**Authors:** Jia-De Cheng, Jing-Yuan Tsai, Chun-Wei Huang, Chien-Hua Wang, Ching-Min Su, Jui-Yuan Chen, Ying-Hao Chu, Wen-Wei Wu

**Affiliations:** † Department of Materials Science and Engineering, 34914 National Yang Ming Chiao Tung University, Hsinchu 30010, Taiwan; ‡ Department of Materials Science and Engineering, 34902 Feng Chia University, Taichung 407, Taiwan; § Department of Materials Science and Engineering, 59215 National United University, Miaoli 360302, Taiwan; ∥ Department of Materials Science and Engineering, 34881 National Tsing Hua University, Hsinchu 300, Taiwan; ⊥ Center for the Intelligent Semiconductor Nano-system Technology Research, National Yang Ming Chiao Tung University, Hsinchu 30010, Taiwan

**Keywords:** high-entropy oxide, resistive random-access memory, in situ TEM, STEM-EELS, resistive switching, structural transformation

## Abstract

High-entropy oxides (HEOs) have emerged as potential candidates for resistive random-access memory (RRAM) owing to their structural robustness and highly tunable electronic configurations. In this study, a transition-metal HEO thin film composed of Ca, Ti, Sr, Ta, Nb, and O was integrated into Au/HEO-based RRAM devices that exhibited a low SET voltage, fast switching speed (30 ns), and long data retention time (10^4^ s). The underlying resistive switching mechanism was uncovered by employing in situ transmission electron microscopy to observe the real-time structural evolution under an electrical bias. A localized monoclinic-to-cubic symmetry transition was observed during the SET process. Energy-dispersive X-ray spectroscopy and electron-energy-loss spectroscopy analyses revealed cationic rearrangements and Ti/Nb redox interactions accompanied by the generation of oxygen vacancies. These results establish the redox–structure-coupled mechanism for understanding resistive switching in complex oxides. In addition, the HEO-based memristors exhibit stable switching characteristics and synaptic plasticity, highlighting their feasibility for next-generation memory and neuromorphic applications.

## Graphical abstract

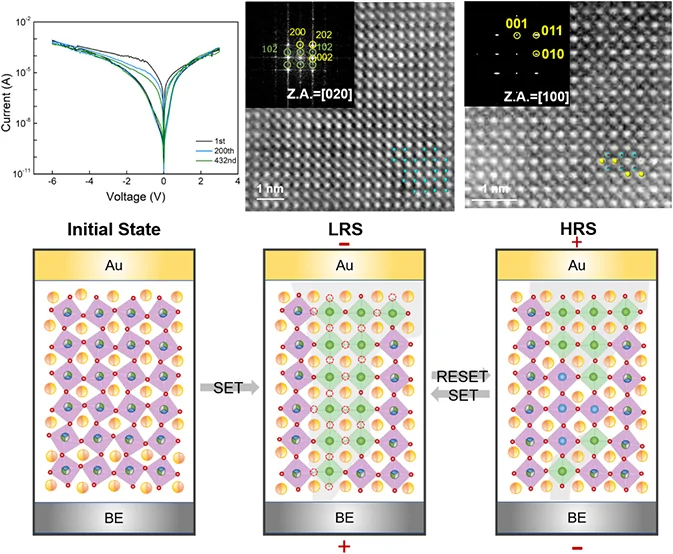

## Introduction

A resistive random-access memory (RRAM) provides distinct features such as exceptional scalability,^1^ low power consumption,^2^ fast switching speed,^3,4^ and simple metal–insulator–metal structure, which makes it a promising candidate for future nonvolatile memory (NVM) technology.^5^ The rapid development of artificial intelligence and neuromorphic computing has resulted in an increased demand for energy-efficient and high-speed NVM. In conventional von Neumann architectures, frequent data transfers between the memory and processors can lead to substantial energy consumption. The growing data workloads limit computing performance because of the von Neumann bottleneck, which results in significant energy loss and latency.^6^ Under such constraints, RRAM has emerged as a potential solution because of its low power consumption and compatibility with in-memory computing.^6–9^

Oxide-based RRAM has been extensively investigated, resulting in the development of various defect-driven switching models.^10,11^ Binary transition-metal oxides such as Nb_2_O_5_,^12^ Ta_2_O_5_,^13,14^ and TiO_*x*_ ^15^ have emerged as the dielectric layers commonly utilized in RRAM devices. In addition to these systems, ABO_3_-type perovskite oxides are recognized for their structural robustness, providing a stable framework for controlled defect formation and electronic modulation.^16–18^ In these systems, resistive switching is commonly associated with oxygen vacancy migration,^19^ cation redox reactions, and localized changes in the electronic structure when inert top electrodes are adopted. Transition-metal cations often exhibit multiple valence states, facilitating charge migration through redox-assisted electronic transport. Under an applied electric field, defects tend to redistribute and aggregate, inducing a reversible metal–insulator transition and the formation of conductive pathways between the top and bottom electrodes. Based on the spatial distribution of defects and the nature of the electrode–oxide interface,^20^ the switching behavior is often described in terms of filamentary and interface-type mechanisms. Based on these observations, various defect-driven switching models have been proposed to describe the resistive switching behavior of oxide-based RRAM devices. Accordingly, most resistive switching models are established under the assumption that a limited number of dominant defect species govern conduction.

High-entropy oxides (HEOs) are multicomponent systems containing several principal cations in near-equimolar ratios, wherein the high configurational entropy contributes to phase stabilization.^21^ Owing to their chemical versatility, structural stability, and defect tolerance,^22^ HEOs have been widely investigated in the fields of energy storage^23^ and hydrogen evolution^24^ for electrocatalytic applications.^25–27^ In these systems, multication redox processes and redox shuttle-like behaviors facilitate charge transfer. These characteristics suggest that HEOs can support charge transport and resistive switching behaviors fundamentally different from those of conventional binary oxides. However, the application of HEOs in RRAM remains relatively underexplored, and the underlying switching mechanisms are not fully understood. Most existing switching models are based on binary or ternary oxides, where the switching mechanism is attributed to the behavior of a single dominant defect, such as oxygen vacancies, metal ions, or grain boundaries. In contrast, the chemically disordered nature and multication redox reactions of HEOs can introduce various conduction pathways that cannot be adequately described by conventional models. Therefore, a comprehensive understanding of how electronic transport, oxygen vacancy dynamics, and redox processes collectively govern the resistive switching behavior in HEO-based RRAM is yet to be systematically elucidated.

To address these issues, HEO-based RRAM devices were fabricated using two bottom electrodes: Au/HEO (Ca, Sr, Ti, Ta, Nb, and O)/SRO/STO and Au/HEO/Nb:STO. Using two bottom-electrode configurations, the effect of the bottom electrodes was systematically assessed, allowing further clarification of the resistive switching behavior associated with the HEO layer. The microstructural evolution was analyzed by high-resolution transmission electron microscopy (HRTEM) and atomic-scale scanning transmission electron microscopy (STEM), which showed transitions from the monoclinic to the cubic phase during resistive switching. In addition, the dynamic switching process was observed using in situ transmission electron microscopy (TEM),^28^ with the diffraction patterns and crystal structures of the HEO layer changing under applied voltage. The changes in composition and valence state were validated using energy-dispersive X-ray spectroscopy (EDS) and electron energy-loss spectroscopy (EELS), which showed a complementary redox relationship among the high-entropy elements observed in the phase-changed regions. The chemically heterogeneous distribution of HEOs leads to element-dependent redox activity and nonuniform defect energetics, which can be coupled with local structural evolution during resistive switching. This study revealed a resistive switching mechanism in HEO-based RRAM that integrates multication redox activity with structural evolution, thereby clarifying how multiple effects are coupled in complex oxide systems. In addition to the defect-structure–property correlations, the device exhibited stable retention characteristics, low SET voltage, and fast switching speed, highlighting its potential as a promising candidate for next-generation memristive devices.

## Results and Discussion

To verify the crystal structure and epitaxial nature of the fabricated devices, the fundamental characterizations of the initial Au/HEO/SrRuO_3_/SrTiO_3_ RRAM devices are shown in Figure 1. The architecture of the Au/HEO/SRO/STO device and corresponding electrical measurement configuration are schematically illustrated in Figure 1a. As shown in Figure 1b, EDS mapping is used to characterize the elemental composition, wherein the different colors represent the corresponding components (Au, Ca, Sr, Ti, Ta, Nb, Ru, and O) and reveal a uniform distribution throughout the layers. Such a homogeneous distribution indicates that the high-entropy perovskite oxide is successfully synthesized, and it is expected to enhance structural stability. The X-ray diffraction (XRD) pattern shown in Figure 1c demonstrates two diffraction peaks at 22.7° and 46.3°. The peak at 22.7° originates from the overlapping contributions of the (020) planes of the monoclinic perovskite-structured HEO and the (001) planes of the cubic perovskite SrTiO_3_, while the peak at 46.3° corresponds to the (002) planes of STO (JCPDS: 74-1296). The presence of sharp diffraction peaks indicates the high crystallinity and epitaxial growth of the HEO thin films on the substrate. An *I–V* curve of the Au/HEO/SRO/STO device is shown in Figure 1d to establish the bipolar resistive switching behavior before the detailed materials analyses. A cross-sectional TEM image of the pristine device is shown in Figure 1e, where the thicknesses of the Au top electrode, HEO dielectric layer, and SRO bottom electrode are ∼30, 60, and 40 nm, respectively. The sharp interfaces between each layer confirm high-quality epitaxial growth. As shown in Figure 1f, the enlarged HRTEM image of the orange dashed region in Figure 1e indicates that HEO dielectric layer is single-crystalline. Through HRTEM and the fast Fourier transform diffraction pattern (FFT-DP), the structure of HEO was further identified to be a monoclinic perovskite structure (space group *P*2_1_/*m*) with a zone axis of [020], which is consistent with the XRD results. The HEO layer undergoes structural distortion driven by octahedral distortion, thereby resulting in crystallization into a monoclinic phase. This behavior is attributed to the existence of multiple B-site cations with different valence states and ionic radii, causing anisotropic distortion of the BO_6_ octahedra.^25,29^ This structural distortion not only lowers the crystal symmetry but may also affect the local electronic structure.

**Figure 1:**
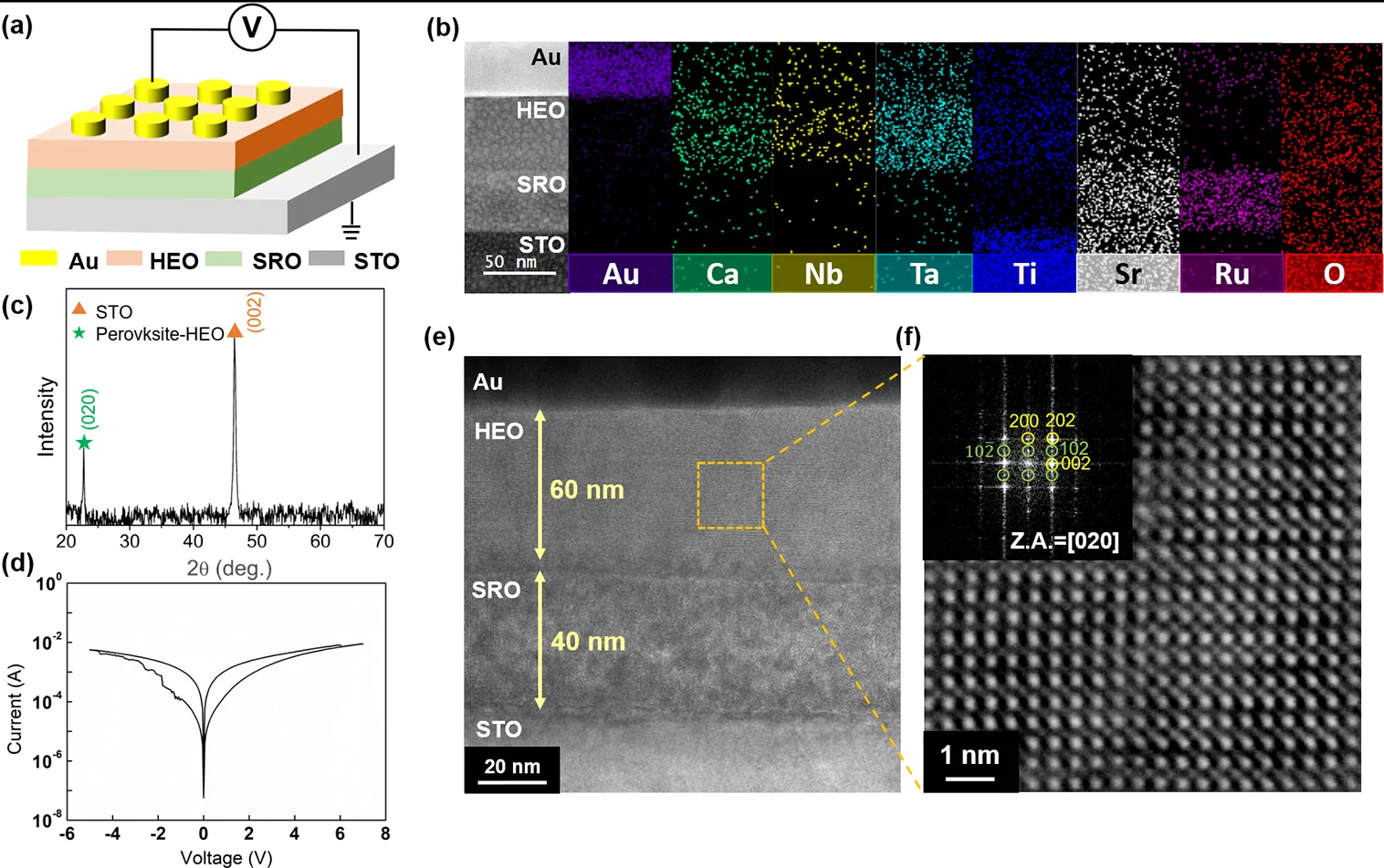
Illustration and basic characterizations of the pristine Au/HEO/SRO/STO device. (a) Schematic of the device. (b) STEM image and EDS mapping results of Au, Ca, Nb, Ta, Ti, Sr, Ru, and O. (c) XRD pattern of perovskite-HEO thin film and STO. (d) *I–V* curve showing the bipolar resistive switching behavior of the Au/HEO/SRO/STO device. (e) Cross-sectional TEM image of the pristine HEO RRAM device. (f) Enlarged HRTEM image and FFT-DP for HEO within the orange dashed region in (e). The yellow and green circles in the FFT-DP represent the main and half-order superlattice reflections, respectively.

Structural evolution is closely correlated with the resistive switching behavior, and therefore, atomic-scale ex situ STEM imaging is performed after repeated SET and RESET operations, as shown in Figure 2. Figure 2a, d, show that the pristine HEO exhibits a monoclinic perovskite structure with a zone axis of [020], which is consistent with the distortion-driven lattice configuration discussed earlier. Upon the application of an external bias, the device is set to a low-resistance state (LRS). As indicated in Figure 2b, a distinct V-shaped region emerges within the HEO layer, indicating a localized structural transformation from the monoclinic to the cubic perovskite phase, as confirmed by the FFT pattern with a zone axis of [100] in Figure 2e, f. The emergence of the V-shaped regions is correlated with oxygen vacancy migration, as is systematically verified in the following analysis. The formation of this cubic region suggests the redistribution of point defects and local relaxation believed to facilitate the structural transition toward a higher-symmetry phase. The cubic regions provide continuous pathways that enhance electronic transport, reducing device resistance. The monoclinic-to-cubic transformation occurs locally within a nanoscale active switching region rather than over the entire device area. The measured electrical response is dominated by the most conductive pathway instead of uniform conduction across the full electrode area. Therefore, nanoscale structural and chemical evolution within the active switching region can significantly modulate the overall device current and resistance when it forms, stabilizes, or disrupts a continuous conductive pathway. When a reverse bias is applied, the device is reset to the high-resistance state (HRS). In the HRS, partial cubic phases are transformed to monoclinic phases (Figure 2g), while some regions retain the cubic structure (Figure 2h). This results in a heterogeneous microstructure characterized by the coexistence of monoclinic and cubic phases within the HEO layer, as illustrated in Figure 2c. The presence of residual cubic regions after RESET suggests that the structural evolution is only partially reversible during resistive switching. RESET process primarily disrupts or suppresses the continuous conductive pathway through oxygen-vacancy redistribution. Therefore, complete restoration of the entire transformed region to the pristine monoclinic structure is not necessarily required for the transition from the LRS to the HRS. The remaining cubic regions may be associated with residual oxygen vacancies and incomplete reversal of Ti/Nb cation redistribution after switching. Lattice rearrangement may be slower and partially stabilized by the local chemical disorder and defect tolerance in the HEO lattice. Thus, the RESET process should be regarded as the rupture of the conductive pathway rather than the full recovery of the pristine lattice.

**Figure 2:**
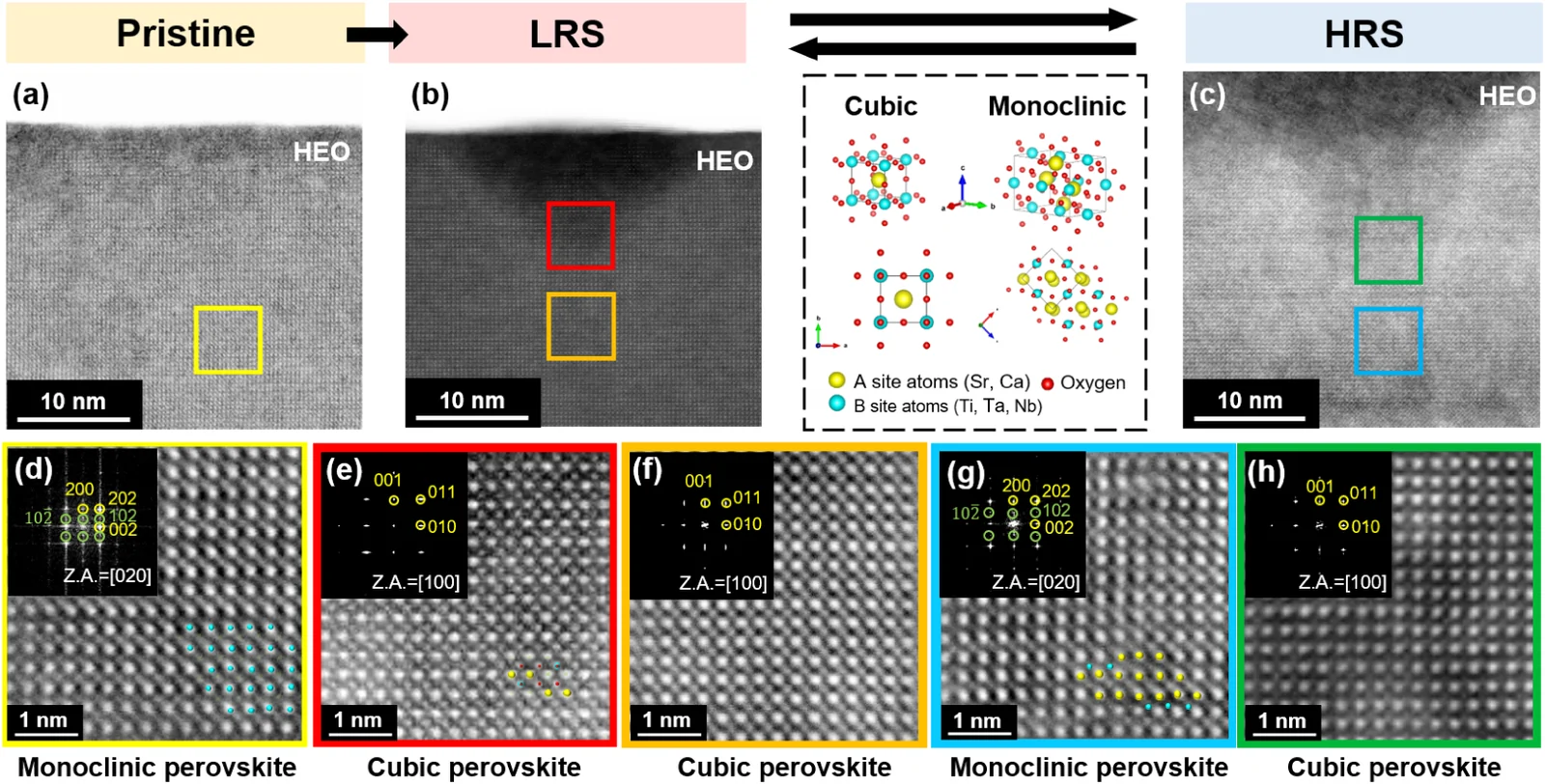
STEM image analysis of the Au/HEO/SRO/STO RRAM device at different resistance states. (a–c) STEM image of the Au/HEO/SRO/STO device at the pristine, low resistance (LRS), and high resistance states (HRS), respectively. (d–h) Enlarged STEM image of monoclinic and cubic phase in perovskite-HEO, corresponding to the positions of the same-colored frame regions in (a–c).

Dynamic structural transformation within the HEO dielectric layer is verified by in situ TEM (Figure 3) to gain deeper insight into the switching process in real time. A series of in situ TEM images extracted from the bias-dependent movie (0 to −2.4 V, see Movie S1), along with the corresponding FFT-DPs shown in the upper right corners, is presented in Figure 3(a–d).

**Figure 3:**
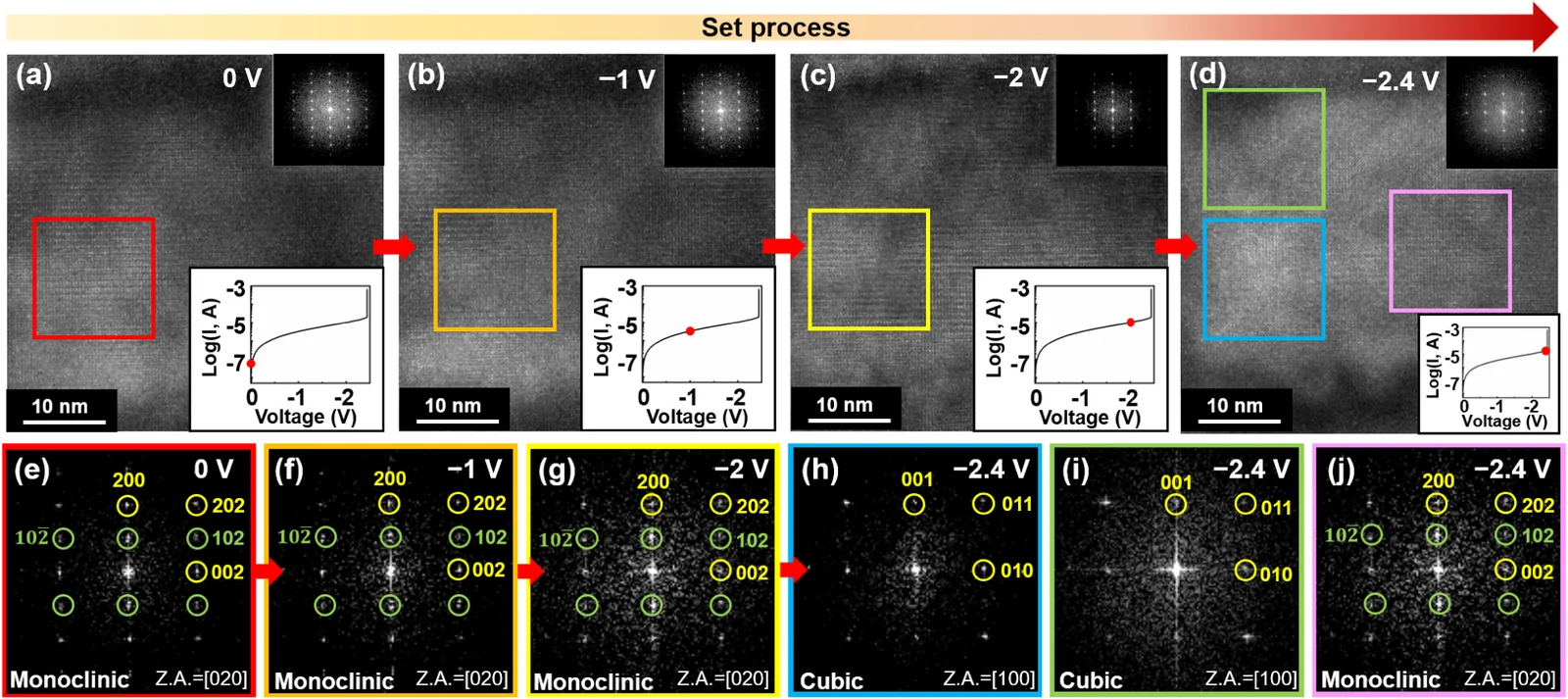
Dynamic observation of structural evolution via in situ TEM. (a–d) A series of in situ TEM images under applied biases of 0 V, −1 V, −2 V, and −2.4 V, respectively, showing the structural evolution from the monoclinic to the cubic phase. (e–g) FFT-DPs obtained from the color-marked regions in (a–c), showing characteristic features of the monoclinic phase under 0 to −2 V bias. (h, i) FFT-DPs from the blue and green-marked regions in (d), indicating the emergence of the cubic phase. (j) FFT-DP from the purple-marked region in (d), suggesting the local retention of the monoclinic phase.

In Figure 3e–j, FFT-DPs are obtained from the corresponding dashed regions of the same colors in Figure 3a–d. Initially, the HEO layer exhibits monoclinic characteristics, with additional superlattice reflections appearing at half-order positions along the vertical reciprocal axis (Figure 3e). In the voltage range of 0 to −2 V (Figure 3e–g), the FFT-DPs retain characteristic features of the monoclinic phase without noticeable changes, suggesting that the monoclinic phase remains dominant in this bias range. Once the bias reached −2.4 V, the characteristic reflections at half-order positions progressively diminish and eventually disappear, as evidenced by the FFT-DP of the overall image shown in Figure 3d. However, structural transformations did not occur throughout the dielectric layer. Instead, localized regions underwent a phase transition with cubic characteristics (Figure 3h and i), while other areas retain their original monoclinic nature (Figure 3j). The current–voltage (*I–V*) curve (Figure S1 a) shows a sharp current increase at −2.4 V, which corresponds precisely to the voltage where the diffraction pattern began to evolve. The abrupt current change near the end of the voltage application is likely a transient response specific to the nanoscale in situ TEM biasing configuration. Because the in situ measurement was performed on a FIB-prepared lamella with different sample geometry, switching volume, and contact configuration from full-device *I–V* measurements, this transient feature should not be directly compared with the *I–V* curves.

In addition to the evolution of the diffraction features observed in the in situ TEM experiment, another in situ observation provides the direct evidence of lattice transformation in real space, as shown in Figure 4. A bias-dependent in situ TEM sequence (0 to −1.65 V, see Movie S2) is shown in Figure 4(a–d), while enlarged HRTEM images and FFT-DPs from the red-dashed regions are presented in Figure 4(e–h). In the initial state (Figure 4e), the pristine HEO layer exhibits a monoclinic perovskite structure in which alternating A-site (Ca/Sr) and B-site (Ti/Ta/Nb) cations form a periodic lattice before any external bias is applied.^30^ The presence of half-ordered reflections and subtle deviations from the cubic symmetry indicate octahedral tilting and anisotropic lattice distortions, which are characteristic of a monoclinic configuration. With the increasing voltage range from 0 to −1.6 V (Figure 4f and g), the lattice distortion is gradually alleviated, suggesting a gradual relaxation of the lattice strain and a continuous transition toward a higher-symmetry configuration. This evolution is accompanied by a weakening of the half-order reflections, which implies a decrease in the octahedral tilt amplitude. Once the bias reaches −1.65 V (Figure 4h), the structure within the red-dashed region transforms into a cubic phase, which is consistent with the crystal structure observed in the LRS during ex situ analyses. The structural evolution observed at −1.65 V represents the onset of local lattice distortion, while the monoclinic-related reflections in the FFT remain visible, indicating an early stage of transformation. In comparison, the disappearance of the half-order reflections at −2.4 V in Figure 3 corresponds to a more developed transformation, where the accumulated lattice evolution becomes sufficiently pronounced to affect the overall FFT features of the selected region. A sudden rise in current is observed when the applied voltage continued to increase to −2.2 V, suggesting the formation of conductive pathways and initiation of resistive switching (Figure S1 b). Further, both in situ experiments based on reciprocal-space and real-space observations showed that the electrical transition occurs near the same critical voltage. This consistency suggests that the onset of the resistive switching process is linked to a field-induced structural transformation. To exclude possible electron-beam-induced structural changes during the in situ TEM observation, a beam-only control experiment was performed under the same imaging conditions without applying electrical biasing, as shown in Figure S2. During 10 min of continuous electron-beam irradiation, both the lattice contrast in the TEM images and the corresponding FFT-DPs retained the monoclinic characteristics, including the half-order reflections along the vertical reciprocal direction (Figure S2g–l). During the electron-beam irradiation, no noticeable structural transformation or disappearance of the monoclinic features was observed, indicating that the monoclinic-to-cubic transformation observed in Figures 3 and 4 is mainly driven by electrical biasing rather than electron-beam effect.

**Figure 4:**
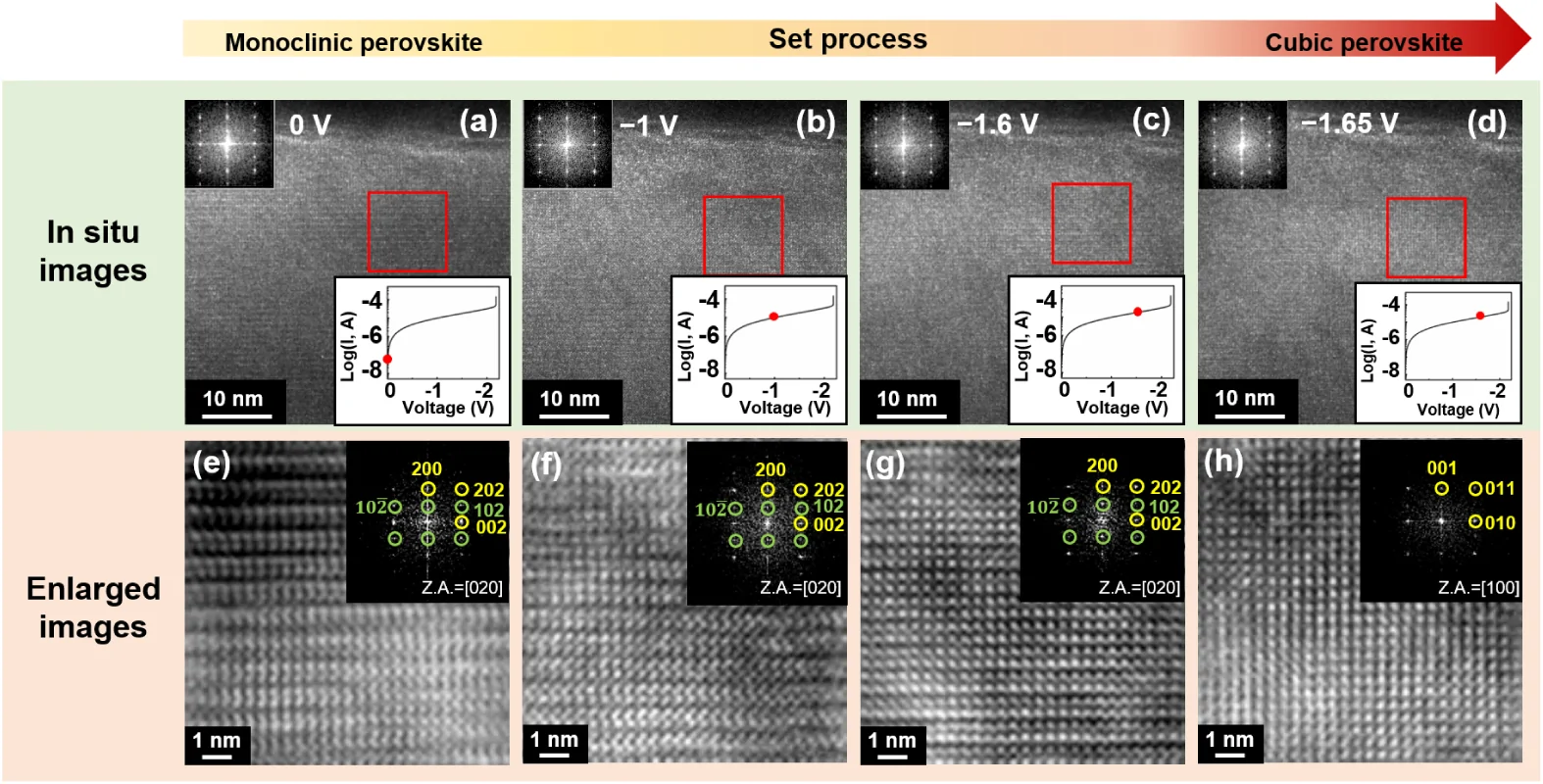
Series of in situ TEM images extracted from the video. (a–d) A series of in situ TEM images under applied biases of 0 V, −1 V, −1.6 V, and −1.65 V, respectively, indicates the structural evolution from the monoclinic to the cubic phase. (e–h) Enlarged HRTEM images and FFT patterns of monoclinic and cubic phases in perovskite-HEO, corresponding to red–framed regions in (a–d).

Spatially resolved EDS and EELS analyses are performed to investigate the redistribution of elements and changes in the valence states and clarify the mechanism underlying the observed structural transformation (Figure 5). The EELS spectra of Ca, Sr, Nb, Ti, and O acquired in the initial state and after electrical biasing are presented in Figure 5a–e. The valence states of the cations are determined by analyzing the fine structure of the L_2,3_ edges, which corresponds to the electronic transitions from the 2p to 3d orbitals. The transition probability is governed by the availability of unoccupied 3d orbitals and their spin–orbit splitting, and therefore, the relative intensities of the L_3_ (2p_3/2_ to 3d) and L_2_ (2p_1/2_ to 3d) white lines provide a sensitive indicator of the electronic configuration. A higher valence state reduces 3d electron occupancy, which enhances transition probabilities and results in a larger integrated L_3_/L_2_ ratio. In the resistive switching process, the observed change in the white-line intensity indicates a redistribution of 3d electron states associated with the redox process and a modification of the local electronic configuration driven by oxygen vacancy migration. According to the EELS profile of the Ca L-edge (Figure 5a), the Ca L_3_-edge peak is located at 348.1 eV, and the L_2_-edge appears at 351.6 eV, respectively, in the initial state. After switching, the values remain almost unchanged at 348.3 and 351.6 eV.^31^ The negligible shift suggests that the local chemical environment and valence state of Ca are preserved during the switching process. Similarly, Figure 5b shows the Sr L_3_-edge at 1946.9 eV and L_2_-edge at 2015.5 eV in the initial state, and the L_3_-edge at 1947.1 eV and L_2_-edge at 2014.7 eV in LRS, respectively.^32^ The absence of significant chemical shifts or splitting for both A-site cations, Ca and Sr, indicates that they retain their divalent oxidation states before and after switching.

**Figure 5:**
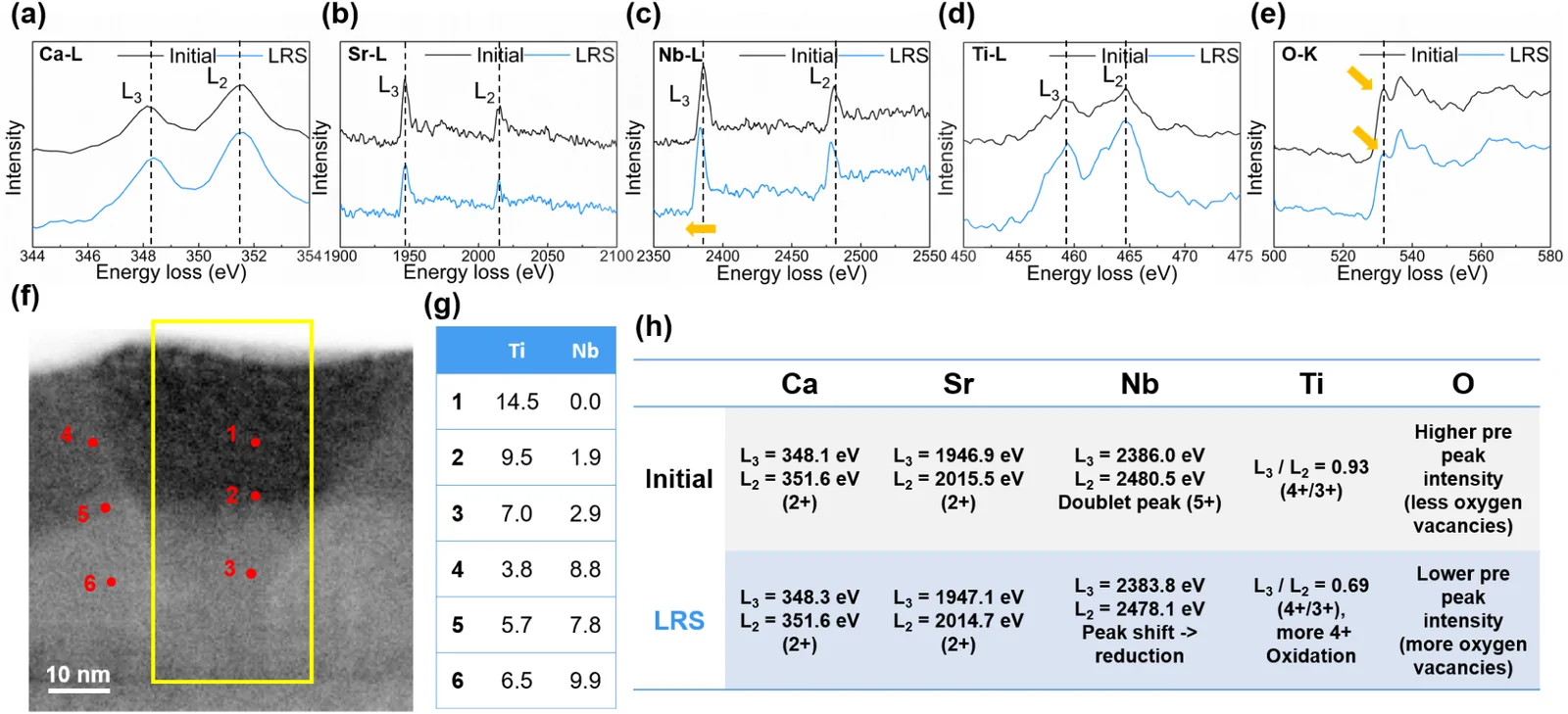
EELS and EDS analysis results of the Au/HEO/SRO/STO RRAM device at different resistance states. (a–e) EELS spectra of the Ca L-edge, Sr L-edge, Nb L-edge, Ti L-edge, and O K-edge acquired from the HEO layer in the initial (black) and cycled (blue) states. (f) STEM image of HEO layer after resistive switching, with marked EDS acquisition points. (g) EDS point results of (f). (h) Summary of EELS results showing the variations in the valence states for the constituent elements between initial state and LRS.

In contrast, the Nb L-edge exhibits a significant chemical shift, as indicated in Figure 5c. In the initial state, the L_3_ and L_2_ edges are located at 2386.0 and 2480.5 eV, and L_2,3_ exhibits a doublet structure characteristic of the Nb^5+^ valence state. After switching, both edges shift toward lower energies (2383.8 and 2478.1 eV), corresponding to a chemical shift of ∼2 eV. This shift is indicative of a reduction process in which Nb^5+^ is partially reduced to Nb^4+^, thereby resulting in a mixed-valence state (Nb^5+^/Nb^4+^) in the LRS.^12,33^ As shown in Figure 5d, the L_3_ and L_2_ peak positions of the initial state of Ti are seen at 459.1 and 464.6 eV, with the Ti-L_3_/L_2_-integrated ratio of 0.93. After switching, the peak positions are observed at 459.3 and 464.6 eV in the LRS, with the Ti-L_3_/L_2_-integrated ratio of 0.69. The Ti-L_2,3_ EELS spectrum exhibits a characteristic intensity distribution, wherein the L_3_ peak is intrinsically weaker than the L_2_ peak. This behavior arises from the limited 3d occupancy and resulting transition probability distribution, wherein the 2p_1/2_ to 3d transition is favored because of the unoccupied density of states. The observed decrease in the L_3_/L_2_ ratio after switching suggests an increased fraction of Ti^4+^ species,^34^ indicating a tendency toward oxidation during the switching process. In addition, both states exhibited crystal-field splitting of the L_3_ edge into two distinct subpeaks corresponding to transitions into the t_2_g and e_g_, states of Ti 3d orbitals. However, because no obvious Ti L-edge peak shift is observed, the Ti spectral evolution should not be interpreted solely as a valence-state change. Local crystal field variations, Ti–O coordination change, and reduced octahedral distortion associated with the structural transformation may also contribute to the Ti L-edge fine structure evolution. Consequently, the observed Ti L-edge change reflects the combined effect of Ti oxidation and local electronic modification in the transformed region. This observation implies that resistive switching is governed not only by a change in the valence states of Ti but also by subtle electronic rearrangements. The concurrent reduction of Nb and oxidation of Ti revealed complementary redox behavior between B site cations. This process establishes a redox shuttle pathway, enabling electron transfer between transition metals to compensate for the local charge imbalance during the resistive switching process. The reduction of Nb^5+^ to Nb^4+^ requires electron injection and is often accompanied by the generation of oxygen vacancies, while the oxidation of Ti is driven by the redistribution of these electrons. As shown in Figure 5e, the O K-edge spectra show prepeaks at ∼531.6 eV and reach a maximum at ∼536.1 eV in both the initial state and LRS. Despite the negligible chemical shift, the prepeak intensity of the LRS weakened with an increase in the oxygen vacancy concentration.^13,35^

Figure S3 presents the O K-edge, Ca L_2,3_-edge, and Ti L_2,3_-edge EELS analyses acquired from the V-shaped region with the corresponding ADF-STEM image marking the regions examined. In Figure S3 a, the V-shaped region is divided into six distinct areas for EELS analysis, where areas 2 and 5 correspond to the transformed region. As shown in Figure S3b, the O K-edge spectra exhibit notable variation in the prepeak features. Region 5, located within the V-shaped conductive pathway, shows the weakest prepeak intensity, with the I_main_/I_pre_ ratio of 4.15. In contrast, regions 4 and 6 positioned outside the V-shaped region reveal lower ratios of 2.38 and 3.22, respectively. The suppressed prepeak feature indicates a higher concentration of oxygen vacancies within the conductive region. The Ca L_2,3_-edge spectra shown in Figure S3c, exhibit negligible chemical shift and nearly unchanged white-line ratios across three regions, suggesting the divalent valence state of Ca^2+^ remains stable during the process. The Ti L_3_/L_2_ ratios of areas 1–6 are 0.68, 0.63, 0.75, 0.73, 0.69, and 0.93, respectively. Notably, areas 2 and 5, located within the V-shaped region, exhibit the lowest L_3_/L_2_ ratio compared to regions on both sides along the horizontal direction, implying a higher proportion of Ti^4+^ species (Figure S3d). The EDS point analyses of the HEO layer after resistive switching operation (Figures[Fig fig5]f and g) are conducted at six positions across the dielectric layer, with points 1 to 3 located inside the V-shaped region and points 4–6 located outside. The quantitative results reveal a clear compositional contrast, and detailed composition is shown in Table S1. The Ti atomic fraction is significantly higher within the transformed region (7.0–14.5 at. %), whereas the Nb concentration is substantially lower (0–2.9 at. %) compared to the surrounding areas (Ti: 3.8–6.5 at. %; Nb: 7.8–9.9 at. %). The distribution suggests a preferential accumulation of Ti accompanied by Nb redistribution in the conductive regions. To provide a pristine compositional baseline, STEM-EDS point analyses were performed on the pristine HEO layer (Figure S4 and Table S2). The pristine HEO shows an average cation-normalized composition of Ca, Sr, Ti, Ta, and Nb of 13.8 ± 1.0, 14.9 ± 0.3, 21.1 ± 2.7, 31.6 ± 2.0, and 18.6 ± 0.3 at.%, respectively. Compared with the baseline, the cycled transformed region exhibits a reduced Nb/Ti ratio, supporting relative Ti enrichment and Nb redistribution associated with the conductive pathway. In addition to the EDS point analyses, the spatial correlation between the transformed region and cation redistribution was further examined by STEM-EDS mapping (Figure S5), where the Ti signal is relatively enhanced within the hourglass-shaped transformed region. EDS analyses were performed on the specimen following the in situ experiment (Figure S6). In (Figure S6a–c), the crystal structure in the LRS state exhibits a cubic perovskite structure with a zone axis of [100], which is consistent with the ex situ observations. As presented in Figure S6d, a noticeable Ti enrichment is observed within the dielectric layer, with a higher atomic fraction (11.1–13.8 at. %) accompanied by a slight Nb redistribution (8.0–9.2 at. %). The relatively moderate degree of Nb redistribution compared to the ex situ results can be attributed to the in situ experiment being involved only a single SET operation, while the ex situ specimen underwent multiple electrical cycles. The pronounced elemental segregation in the ex situ specimen is attributed to the cumulative ionic migration driven by repeated switching cycles. However, even under a single SET operation, the in situ specimen already exhibited the characteristic tendency of Ti enrichment and Nb redistribution. Correlated with the EELS findings, where Ti exhibits an increased proportion of Ti^4+^ and Nb shows a reduction from pentavalent to tetravalent ions, these observations support a complementary Ti–Nb redox process. Collectively, spatially coupled compositional and electronic variations indicate that resistive switching is governed by cation redistribution and local charge compensation, driven by oxygen-vacancy migration within the V-shaped conductive region. Thus, although the localized region suggests a filamentary switching process, it should not be interpreted as a purely classical oxygen-vacancy filament. Instead, the switching behavior in the HEO memristor is better described as a structurally reconstructed conductive pathway governed by coupled defect-cation-structure evolution, rather than by a purely oxygen-vacancy-only filament mechanism.

An additional HEO device employing Nb:STO as the bottom electrode (hereafter referred to as the Nb:STO-BE device) was fabricated to verify that the resistive switching mechanism was intrinsic to the HEO dielectric layer rather than dominated by the specific bottom electrode. We first characterized the pristine structure of the Au/HEO/Nb:STO device to confirm that the crystal structure and epitaxial relationship of the HEO film were consistent with those of the Au/HEO/SRO/STO device. The HEO layer had a thickness of ∼40 nm and exhibited a monoclinic perovskite structure along the [020] zone axis, which was consistent with the HEO device employing SRO as the bottom electrode (referred to as the SRO-BE device) (Figure S7a–c). As shown in Figure S7d, the EDS mapping results demonstrate uniform elemental distribution of all constituent elements within the HEO layer, confirming the homogeneous compositional nature of the thin film. Subsequently, electrical cycling measurements were performed, and the device was switched to the LRS. A similar filament-like region emerges in the LRS state, and the same monoclinic-to-cubic transformation is observed. In Figure S8a, an hourglass-shaped region emerges in the cycled HEO layer, which is presumed to be the conductive pathway. A distinct phase boundary is observed in Figure S8c. The right-hand region exhibits a cubic perovskite structure (Figure S8f), while the left-hand region is identified as a tetragonal perovskite phase (Figure S8e). Perovskite oxides are known for their structural flexibility under external energy inputs such as heating or electrical bias, and their degree of octahedral tilting and lattice distortion is reduced progressively. As illustrated in Figure S8b, the tetragonal phase acts as an intermediate configuration in the structural evolution of the perovskite oxides.^36,37^ In this study, a series of similar transformations is observed in the HEO layer (Figure S8d–f), where the emergence of the intermediate phase provides additional evidence supporting the progressive structural transformation from the monoclinic to the cubic phase.^38^ This result confirms that the substitution of the bottom electrode does not change the structural evolution pathway of the HEO layer during the switching process. However, variations in the electrical properties may still arise because of electrode-dependent effects.

Figure 6 shows the RRAM characteristics of the Au/HEO/SRO/STO and Au/HEO/Nb:STO devices. The current–voltage (*I–V*) curves of the SRO-BE device are measured in the DC voltage sweep mode, containing the first (black curve), 100th (blue curve), and 180th (green curve) sweep cycles (Figure 6a). The voltage sweep sequence is applied as 0 V → −5 V → 0 V → +7 V → 0 V. The SRO-BE device is set to the LRS via negative bias and reset to the HRS via positive bias, exhibiting bipolar characteristics. As indicated in Figure 6b, the *I–V* curve of the Au/HEO/Nb:STO device exhibits bipolar characteristics. The black, blue, and green curves represent the first, 200th, and 432nd sweep cycles, respectively. The device is set to LRS via positive bias, and reset to HRS via negative bias, with the sweep sequence as 0 V → +3 V → 0 V → −6 V → 0 V. The on/off ratio of the SRO-BE device was ∼20, whereas that of the Nb:STO-BE device is ∼140. As shown in Figure 6g, the cumulative probability distributions of the HRS and LRS for both devices are measured at a reading voltage of 0.1 V. The coefficient of variation (σ/μ) for each resistance state is calculated to evaluate the switching uniformity of the two devices. The Nb:STO-BE device exhibits smaller values (0.15 for HRS and 0.63 for LRS) compared with those of the SRO-BE device (0.57 for HRS and 0.74 for LRS), indicating narrower resistance distributions in both states. The Nb:STO-BE device demonstrates better operational stability based on cumulative probability distributions and cycling performance. Although the substitution of bottom electrodes does not alter the structural evolution associated with resistive switching, it may have influenced the reliability. As shown in Figure S9, the lattice of the SRO layer became distorted after repeated cycling. In the pristine state, the TEM images revealed that the SRO layer exhibited a cubic perovskite structure (Figure S9a and b). After electrical cycling, numerous locally distorted grains appeared in the SRO layer (Figure S9c and d). The EDS mapping results further revealed a noticeable decrease in the oxygen concentration (Figure S9e), which suggests that oxygen from the SRO layer participated in the migration process during resistive switching,^39^ which is associated with the relatively low oxygen-vacancy formation energy of SRO.^40^ Under repeated bidirectional biasing, the continuous migration of oxygen ions across the HEO/SRO interface likely contributes to the accumulated lattice distortion and reduced stability observed in the SRO-BE devices.^41^ Considering its enhanced stability and narrower resistance distribution, the Nb:STO-BE device is adopted for subsequent electrical characterization. The resistance-state retention ability is measured for more than 10^4^ s, as shown in Figure 6c. As shown in Figure 6d, the resistances of the HRS and LRS are extracted under a constant reading voltage (0.1 V) over 250 cycles. The results exhibit a resistance window of approximately 1 order of magnitude between the HRS and LRS, which is consistent with the value of the on/off ratio in the cycling test. Electrical measurements are conducted on ten individual devices to ensure device-to-device consistency (Figure 6e). The ten devices show distinguishable HRS and LRS, but noticeable device-to-device variation remains. Further, the transient current responses under a voltage pulse were applied to the device. A positive voltage pulse (2 V) induced a sharp current spike within ∼30 ns, demonstrating the high switching speed of the SET process (Figure 6f). In contrast, during the RESET process, a negative voltage pulse (−2 V) induces an abrupt current drop within ∼100 ns (Figure 6h). The synaptic plasticity characteristics of the HEO-based RRAM device are measured to assess its potential for neuromorphic computing applications, as indicated in Figure S10. The long-term potentiation and depression (LTP/LTD) behaviors are measured under 100 consecutive voltage pulses (Figure S10a). Under consecutive pulses, the results exhibit an incremental conductance trend. Short-term synaptic plasticity is demonstrated by paired-pulse facilitation (PPF), and the PPF index decreases with increasing intervals following an exponential decay fitting curve. The spike-timing-dependent plasticity characteristics are also emulated, in which the top and bottom electrodes mimic the pre- and postsynapses (Figure S10c). A positive synaptic weight change is observed when the presynaptic spike precedes the postsynaptic spike, while a negative synaptic weight change is observed when the spike order is reversed. These results indicate that the HEO-based RRAM device can emulate key synaptic plasticity behaviors, supporting its potential application in neuromorphic computing. In addition to the synaptic plasticity measurements, the *I–V* curve analyses at varying sweep rates and temperatures are provided in Figure S11. The results reveal that higher sweep rates lead to slightly reduced on/off ratios (Figure S11a). Across a range of temperatures of 20, 50, 80 °C, and then back to 20 °C, the resistance values of both states decrease moderately with temperature, indicating the semiconducting-like nature of conductive pathways,^21,42^ as indicated in Figure S11b and c. In particular, the decrease in HRS resistance with increasing temperature may be associated with enhanced ionic defect mobility. However, no abrupt thermally induced switching or drastic resistance change is observed in the measured temperature range, indicating that external temperature does not drive the structural transformation.

**Figure 6:**
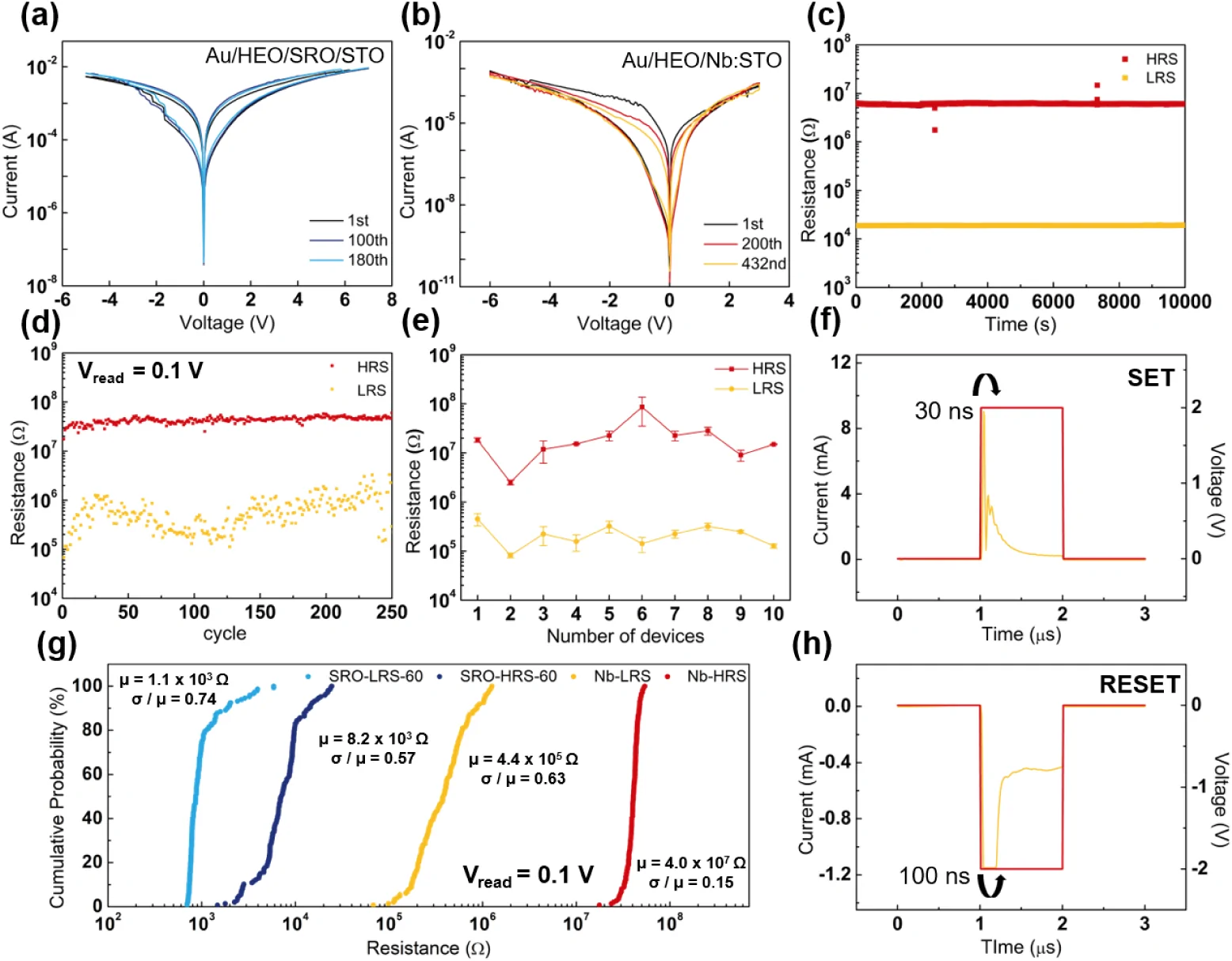
Electrical properties of the Au/HEO/SRO/STO and Au/HEO/Nb:STO devices. (a) *I–V* curves of the Au/HEO/SRO/STO device for 180 cycles. (b) *I–V* curves of the Au/HEO/Nb:STO device for 432 cycles. (c) Retention measurements for the HRS and LRS of the Au/HEO/Nb:STO device. (d) Endurance measurements of the Au/HEO/Nb:STO device at the reading voltage of 0.1 V. (e) Distributions of the HRS/LRS from ten devices with Nb:STO bottom electrodes. (f) Pulse test for the SET process of the Au/HEO/Nb:STO device. (g) The cumulative probabilities at the reading voltage of 0.1 V for two different bottom electrodes, SRO/STO and Nb:STO. (h) Pulse test for the RESET process of the Au/HEO/Nb:STO device.

The *I–V* curve fitting results are analyzed to clarify the conduction mechanism underlying the resistive switching behavior, as shown in Figure S12a. The *I–V* curves are first replotted in ln­(*I*)–ln­(*V*) coordinates, and the corresponding slopes are used to distinguish between multiple transport regimes (Figure S12b and c). During the SET process, the HRS follows a trap-controlled space-charge-limited conduction mechanism, wherein the *I–V* relationship evolves through three characteristic regions.^43,44^ At low electric fields, the slope of ∼1.3 indicates an Ohmic conduction (*I*∝*V*), where thermally excited free carriers dominate electron transport.^45^ As the applied voltage increases, the injected carriers begin to occupy the trap states in the HEO layer, giving rise to a higher slope (∼2.58) corresponding to the trap-filled-limited transition. Once most traps are filled, the current exhibits a quadratic dependence on voltage (*I* ∝ *V* ^2^), representing the Mott–Gurney law regime, where the transport is dominated by space charge. In the higher electric field region, the slope increases to ∼4.01, as the injected electrons gradually occupied oxygen vacancy-related traps, reducing the resistance and leading to a transition from the HRS to the LRS. Following the SET process, the LRS conduction behavior exhibits near-Ohmic characteristics, indicating that carrier transport occurs through a lower-resistance conductive pathway. During the RESET process, the LRS followed Ohmic-like transport. As the applied reverse bias increases, the conductive pathway is disrupted because of the redistribution of oxygen vacancies, which leads to rupture. After this rupture, the device returned to the HRS, and the conduction behavior followed the trap-controlled SCLC-like behavior, strongly nonlinear behavior with slopes larger than 2. To further examine the electrode-area dependence of switching behavior, devices with top-electrode diameters of 80 and 200 μm were measured (Figure S13). Both devices exhibit bipolar resistive switching behavior, and the measured currents remain within the same order of magnitude. This result indicates that the switching current is not simply governed by homogeneous conduction across the entire electrode area, but is more consistent with localized conductive-pathway formation.

Based on the combined results of electrical transport analysis and microscopic characterization, a correlation emerged between the oxygen vacancy dynamics, local cation valence changes, and reversible modulation of conduction pathways. A schematic is provided in Figure 7 to summarize the mechanism underlying the SET and RESET transitions. In the initial state, the HEO dielectric layer exhibits a uniformly ordered monoclinic perovskite structure with homogeneous elemental distribution and no observable structural defects (Figure 7a). Based on the voltage configuration, the migration direction of oxygen vacancies and the corresponding redox evolution are discussed with respect to the biased Au top electrode and the grounded bottom electrode. During the SET process (Figure 7b), under a negative bias applied to the Au top electrode, an electric field-induced complementary redox reaction occurs at the B sites.^46^ The substitutional reaction involves the partial oxidation of Ti from Ti^3+^ to Ti^4+^ and the partial reduction of Nb from Nb^5+^ to Nb^4+^. Concurrently, oxygen vacancies are generated through the electron shuttle process,^47^ migrated toward the top electrode, and gradually accumulated to form conductive pathways. In the conductive regions, an increased Ti concentration suppresses the monoclinic octahedral distortion of the perovskite lattice. According to the Goldschmidt tolerance factor (*T*),^48^ an ideal cubic perovskite compound has *T* = 1. In contrast, compounds with *T* < 1 tend to adopt lower-symmetry structures involving octahedral tilting and lattice distortion because of inefficient ionic packing. The enrichment of Ti because of its smaller ionic radius relative to other B-site cations effectively decreases the average B-site radius and promotes a transition toward a more symmetric cubic perovskite structure. The A-site cations, Ca and Sr, remain in their divalent states throughout the SET process, preserving the perovskite framework and not contributing to redox-driven conduction. We examine the intrinsic bonding energetics and vacancy formation thermodynamics of the constituent B-site cations to rationalize why Ti and Nb actively engage in field-induced redox reactions, while Ta remains chemically inert. The oxygen vacancy formation energies^49–51^ and metal–oxygen bond dissociation energies (BDEs) provide critical insight into their different redox propensities. Reported first-principles calculations show that Ti-based oxides possess the lowest E_vac_, which makes Ti the most susceptible to reacting with oxygen. Nb exhibits intermediate E_vac_ values, enabling partial reduction in the conductive regions.^51^ In contrast, Ta possesses a high E_vac_,^51,52^ which reflects the exceptional stability of the Ta–O bond and its strong resistance to oxygen deficiency. For completeness, the trend is consistent with the BDEs (Ta–O (839 kJ/mol) > Nb–O (726.5 ± 10.6 kJ/mol) > Ti–O (666.5 ± 5.6 kJ/mol)),^53^ which supports the greater redox flexibility of Ti and Nb compared to that of Ta. Based on this, we proceed with the RESET transition. When a reverse bias is applied, the system undergoes a RESET process (Figure 7c). The previously accumulated oxygen vacancies migrate away from the conductive regions, and B-site cations, especially Ti and Nb, restore their original valence states through a reverse redox reaction (Ti^4+^ → Ti^3+^, Nb^4+^ → Nb^5+^). The disruption of the vacancy-rich channels and recovery of the monoclinic lattice revert the device to the HRS. Resistive switching behavior arises from the reversible interaction between the oxygen vacancy dynamics and resulting lattice symmetry changes, which concurrently modulate the conductive pathways. The Ti/Nb redox activity and oxygen vacancy accumulation locally enhanced the symmetry and created conduction paths during the SET process, while vacancy redistribution and valence restoration recovered the monoclinic framework during the RESET process. The high-entropy environment provides the structural adaptability required for this reversible transformation, whereas Ta serves as a chemically stable anchor that suppresses uncontrolled reduction. The relationship between the monoclinic-to-cubic transformation and conductive pathway formation can be understood as a coupled defect-redox-structure evolution. During the SET process, the applied electric field drives oxygen-vacancy migration and local charge compensation, resulting in oxygen deficiency within the switching region. The accumulation of oxygen vacancies enhances electronic conduction and promotes the formation of a localized conductive pathway. The altered local chemical environment is accompanied by Ti enrichment and Nb redistribution. Ti enrichment within the transformed region may reduce the octahedral distortion, facilitating the transition toward a higher-symmetry cubic structure. Thus, the observed monoclinic-to-cubic transformation should not be regarded as the sole origin of resistance switching. Compared with conventional binary-oxide VCM systems, the HEO system offers greater freedom in materials design due to its compositional complexity. The selection and combination of multiple cations can be used to tune redox chemistry, cation-size mismatch, octahedral distortion, and phase stability in a coupled manner. Hence, the chemical complexity of the HEO should not be regarded merely as an additional complex oxide, but rather as a design platform for engineering defect-cation-structure coupling during resistive switching.

**Figure 7:**
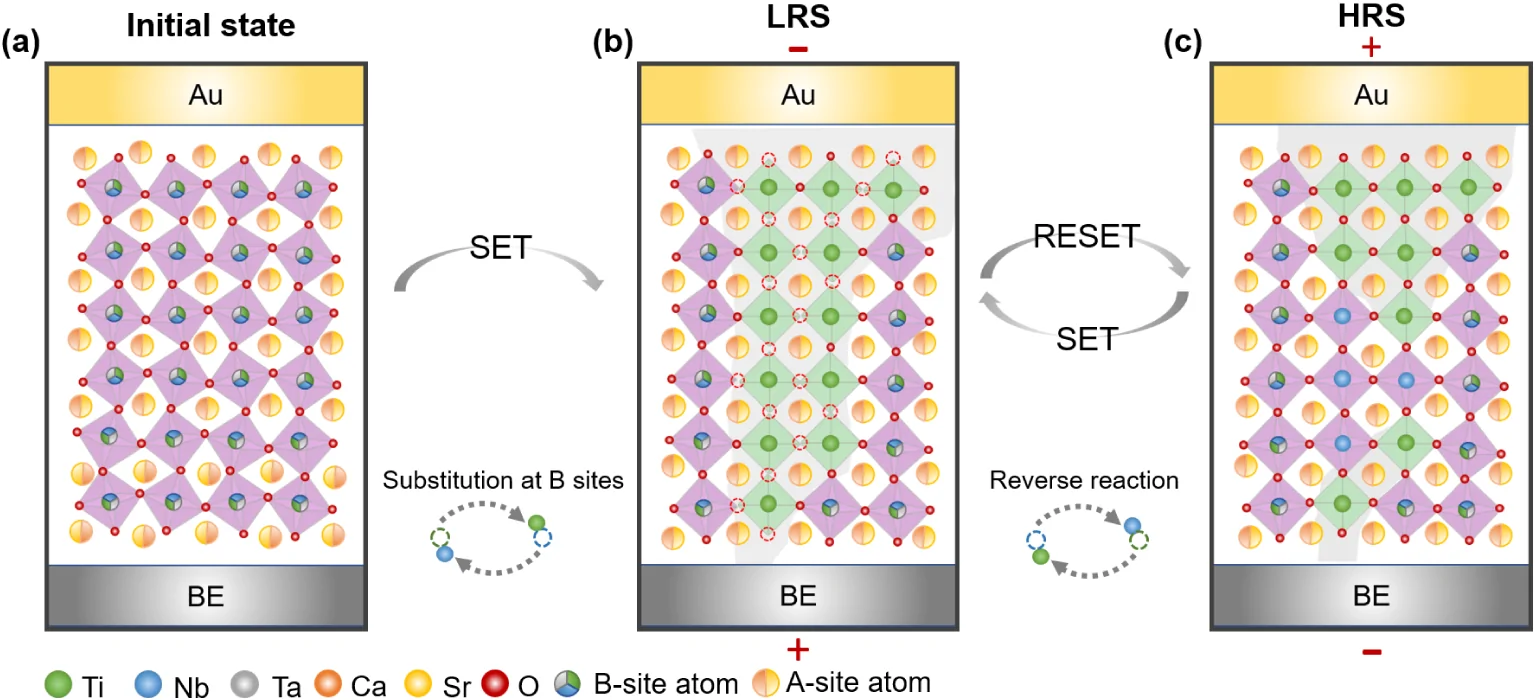
Schematic of the RS process of the HEO-based device. (a) Initial state of the device with evenly distributed B-site cations (Ti, Nb, and Ta) and A-site cations (Ca and Sr) in the perovskite lattice. (b) Under an applied bias, a substitutional redox reaction occurs between Ti and Nb, accompanied by the generation of oxygen vacancies. (c) Under a reverse bias, the redox reaction is reversed, restoring the original valence states of the cations and disrupting conductive paths.

## Conclusions

We reported HEO-based memristors that exhibit resistive switching behavior attributed to the coupling effect between oxygen vacancy dynamics, multication activity, and lattice symmetry evolution. During the HRS-to-LRS transition, a monoclinic-to-cubic structural transformation was directly observed using in situ TEM analysis. EDS and EELS analyses revealed that the chemically disordered HEO framework enabled element-dependent redox activity with a complementary redox behavior between Ti and Nb. Oxygen vacancy migration driven by the electric field initiates electron redistribution, wherein Ti and Nb reorganize to accommodate vacancy accumulation or depletion and restore lattice symmetry. Meanwhile, Ta acts as a stabilizing anchor, while A-site cations (Ca/Sr) maintain stable divalent states to form the structural backbone. During the SET process, the HEO transforms from the monoclinic phase to the cubic phase owing to Ti accumulation, facilitating a low-resistance state. Unlike conventional binary oxide systems, redox-defect-structure coupling effects provide a dynamic pathway for lattice symmetry modulation and the formation of conductive paths during resistive switching. Furthermore, HEO-based memristors exhibited stable resistive switching properties and synaptic plasticity, demonstrating the feasibility of HEOs for next-generation memory applications. This work not only establishes the mechanism of resistive switching but also provides insights into the relationship between lattice symmetry and multication redox interactions in complex oxides.

## Methods

### Fabrication of the Au/HEO/SRO/STO and Au/HEO/Nb:STO Devices

Two types of RRAM devices, Au/HEO/SrRuO_3_/SrTiO_3_ (Au/HEO/SRO/STO) and Au/HEO/Nb-doped SrTiO_3_ (Au/HEO/Nb:STO), were fabricated. For the Au/HEO/SRO/STO configuration, a 40 nm-thick SRO bottom layer was first deposited on (001)-oriented STO substrate via pulsed laser deposition (PLD) using a KrF excimer laser (λ = 248 nm) at 680 °C under an oxygen partial pressure of 100 mTorr. Subsequently, a HEO thin film with a nominal composition (Ca, Sr, Ti, Ta, Nb, and O) was deposited on the top of the SRO layer using PLD at an oxygen partial pressure of 160 mTorr and a temperature of 700 °C. For the Au/HEO/Nb:STO configuration, the HEO thin film was grown directly on (001)-oriented Nb:STO substrates under the same conditions. Au top electrodes were deposited onto the HEO surfaces of both device types via E-gun evaporation at a deposition rate of 0.5 Å/s using a 200 μm-diameter shadow mask, corresponding to an electrode area of approximately 3.14 × 10^–4^ cm^2^, as shown in Figure S15b.

### Measurements of Electrical Properties

The DC *I–V* characteristics of the devices were measured using an Agilent 4145B semiconductor parameter analyzer at room temperature and at controlled temperatures of 20, 50, and 80 °C. Pulse measurements were performed using an Agilent B1500A semiconductor parameter analyzer. During all electrical measurements, a bias voltage was applied to the Au top electrodes, while the SRO and Nb:STO bottom electrodes were grounded. Therefore, the voltage polarity described in this work refers to the potential of the Au top electrode with respect to the grounded bottom electrode.

### Materials Characterization

Cross-sectional TEM and STEM analyses were conducted for examining the microstructural evolution of the HEO dielectric layer in the initial state, LRS, and HRS. The lamellar TEM specimens were prepared using a focused ion beam system (Zeiss CrossBeam 350). The thickness of the specimens was controlled below 100 nm to confirm high-resolution imaging, and the detailed preparation process is shown in Figure S15d–g. A platinum layer was deposited on the Au surface to protect the target area, and both sides of the lamellae were milled to thin the specimen. Subsequently, the as-prepared lamellae were lifted and transferred onto Cu grids using glass tips. The TEM images were acquired using a field-emission TEM (JEOL JEM-F200) operated at an accelerating voltage of 200 kV. An EDS (Oxford EDS 100 TLE system) integrated into the TEM was used for elemental analyses. ADF-STEM imaging was performed using a Cs-corrected STEM instrument (JEOL, ARM-200F) operated at 200 kV, and valence state analysis was conducted using EELS (Gatan Quantum 965) integrated into the system. For in situ specimen preparation, the as-fabricated lamella was transferred to an e-chip, and platinum wires were deposited on the top and bottom electrodes, enabling a perpendicular current to flow through the sample, as indicated in Figure S15a and b. Subsequently, the e-chip was placed on a ProtoChips Aduro 300 in situ holder (Figure S15h).

## Supplementary Material







## Notes

Movie S1: The sample was biased from 0 to −2.4 V during the in situ TEM experiment. Initially, the HEO layer exhibited monoclinic characteristics, as evidenced by the presence of half-order reflections. As the applied voltage increased to −2.4 V, the half-order reflections gradually disappeared, indicating a structural transition from the monoclinic to a cubic phase (AVI)
Movie S2: The sample was biased from 0 to −1.65 V during the in situ TEM experiment, enabling direct observation of the structural evolution in real space. Initially, the observed region exhibited monoclinic characteristics. As the applied voltage increased to −1.65 V, the lattice structure gradually evolved toward cubic characteristic. (AVI)
Details of the *I–V* curves; STEM images; EDS point analyses; experimental setup for in situ experiments; details of the device fabrication process and the preparation of in situ TEM samples; pulse and variable-temperature measurements of RRAM devices; other analyses of the results (PDF)
