# Supplementary material for: In Situ Transmission Electron Microscopy Observation of Redox–Structure-Coupled Resistive Switching in High-Entropy Oxide Memristors
Source: ACS Nano. 2026 Aug 26;20(35):24479–91. doi: 10.1021/acsnano.6c09072 (PMC13564968; doi:10.1021/acsnano.6c09072)
Supplement: Supplementary file 1 [file nn6c09072_si_003.pdf]

Supporting Information

In Situ Transmission Electron Microscopy Observation of Redox–Structure Coupled Resistive Switching in High-Entropy Oxide Memristors

Jia-De Cheng[1], *Jing-Yuan Tsai*[1], *Chun-Wei Huang*[2], *Chien-Hua Wang*[1], *Ching-Min Su*[1], *Jui-Yuan Chen*[3], *Ying-Hao Chu*[4], *Wen-Wei Wu*[1, 5, *]

[1]Department of Materials Science and Engineering, National Yang Ming Chiao Tung University, Hsinchu 30010, Taiwan

[2]Department of Materials Science and Engineering, Feng Chia University, Taichung, 407, Taiwan

[3]Department of Materials Science and Engineering, National United University, Miaoli 360302, Taiwan

[4]Department of Materials Science and Engineering, National Tsing Hua University, Hsinchu, 300, Taiwan

[5]Center for the Intelligent Semiconductor Nano-system Technology Research, National Yang Ming Chiao Tung University, Hsinchu 30010, Taiwan

[*]Correspondence and requests for materials should be addressed to W.-W.W (email: wwwu@nycu.edu.tw)

List of contents

Table S1 | Detailed EDS points results of the cycled HEO layer in Figure 5(f).

Table S2 | Cation-normalized composition of pristine and cycled HEO regions obtained from EDS point analyses.

Figure S1 | Corresponding *I–V* curves of in situ experiments (a) 1 and (b) 2.

Figure S2 | Electron-beam irradiation control experiment without electrical bias.

Figure S3 | O K-edge, Ca L_{23}-edge, and Ti L_{23}-edge EELS spectra analysis of cycled HEO dielectric layer.

Figure S4 | STEM images and EDS point analyses of the pristine HEO layer.

Figure S5 | STEM image and EDS mapping results of Ca, Nb, Ta, Ti, Sr, and O.

Figure S6 | STEM images and EDS points analysis of the HEO layer after the in situ experiment.

Figure S7 | Basic characterizations of the pristine Au/HEO/Nb:STO device.

Figure S8 | Illustration and STEM images of the structural transformation of perovskite-HEO.

Figure S9 | TEM images and EDS maps of the initial and cycled SRO layer.

Figure S10 | Synaptic Plasticity Characteristics of HEO-based RRAM devices.

Figure S11 | Electrical properties of Au/HEO/Nb:STO device at varying sweep rates and temperatures.

Figure S12 | Electrical measurements of the transport behavior of the device.

Figure S13 | Electrical properties of Au/HEO/Nb:STO device at different electrode sizes.

Figure S14 | Process for fabrication of Au/HEO/SRO/STO and Au/HEO/Nb:STO RRAM devices.

Figure S15 | In situ TEM lamella specimens prepared using the focus ion beam system.

Table S1. Detailed EDS points results of the cycled HEO layer in Figure 5(f).

At %	Point 1	Point 2	Point 3	Point 4	Point 5	Point 6
**O**	46.9	50.2	44.1	47.6	48.3	42.9
**Ca**	4.8	3.6	5.3	4.6	3.1	8.1
**Ti**	14.5	9.5	7.0	3.8	4.6	5.7
**Sr**	7.8	15.6	24.0	11.7	20.2	17.4
**Nb**	0.0	1.9	2.9	8.8	5.5	7.8
**Ru**	1.4	0.0	0.6	1.1	0.0	0.0
**Ta**	20.1	19.2	15.8	22.4	17.3	17.5
**Au**	4.6	0.0	0.2	0.0	1.1	0.6

Table S2. Cation-normalized composition of pristine and cycled HEO regions obtained from EDS point analyses.

Region	Label	Ca	Sr	Ti	Ta	Nb	Nb/Ti
Pristine state	Point 1	13.8	15.2	20.4	31.9	18.7	0.9
	Point 2	14.8	14.8	18.9	33.3	18.2	1.0
	Point 3	12.9	14.7	24.1	29.5	18.8	0.8
	Pristine Avg.	13.8±1.0	14.9±0.3	21.1±2.7	31.6±2.0	18.6±0.3	0.9±0.1
Inside V-shape	Point 4	10.2	16.5	30.7	42.6	0.0	0.0
	Point 5	7.2	31.6	19.0	38.4	3.8	0.2
	Point 6	9.6	43.6	12.7	28.7	5.3	0.4
Outside V-shape	Point 7	9.0	22.8	7.4	43.7	17.2	2.3
	Point 8	6.1	39.8	9.1	34.1	10.8	1.2
	Point 9	15.0	32.3	5.8	32.5	14.5	2.5

The cation compositions were normalized to the total Ca + Sr + Ti + Ta + Nb content. Oxygen and minor signals from Au, Pt, and Ru were excluded from the normalization. Values are reported in cation-normalized at.% and rounded to one decimal place. Average values are reported as mean ± standard deviation where applicable. Points 1-3 correspond to the pristine baseline in Figure S4. Points 4-6 and Points 7-9 correspond to the inside and outside regions of the V-shaped transformed region, respectively.

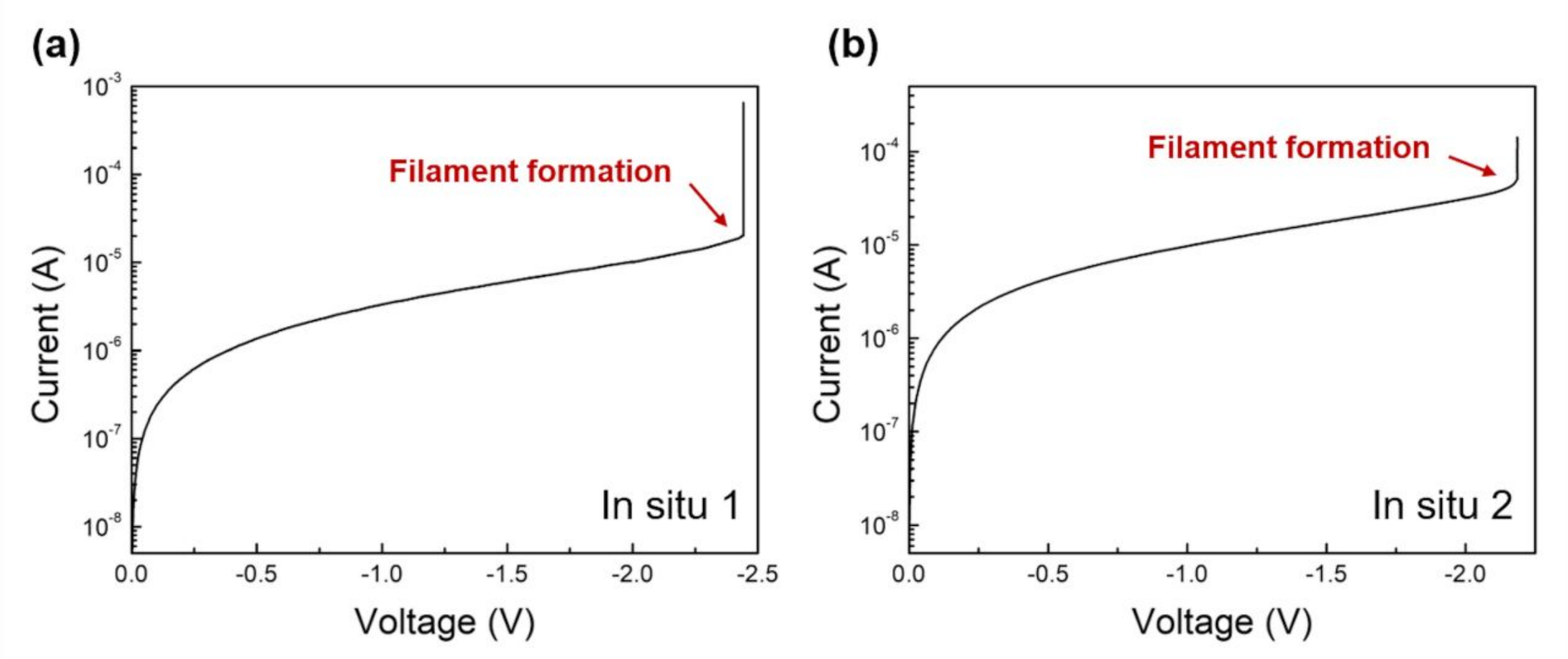

Figure S1. Corresponding *I–V* curves of in situ experiments (a) 1 and (b) 2.

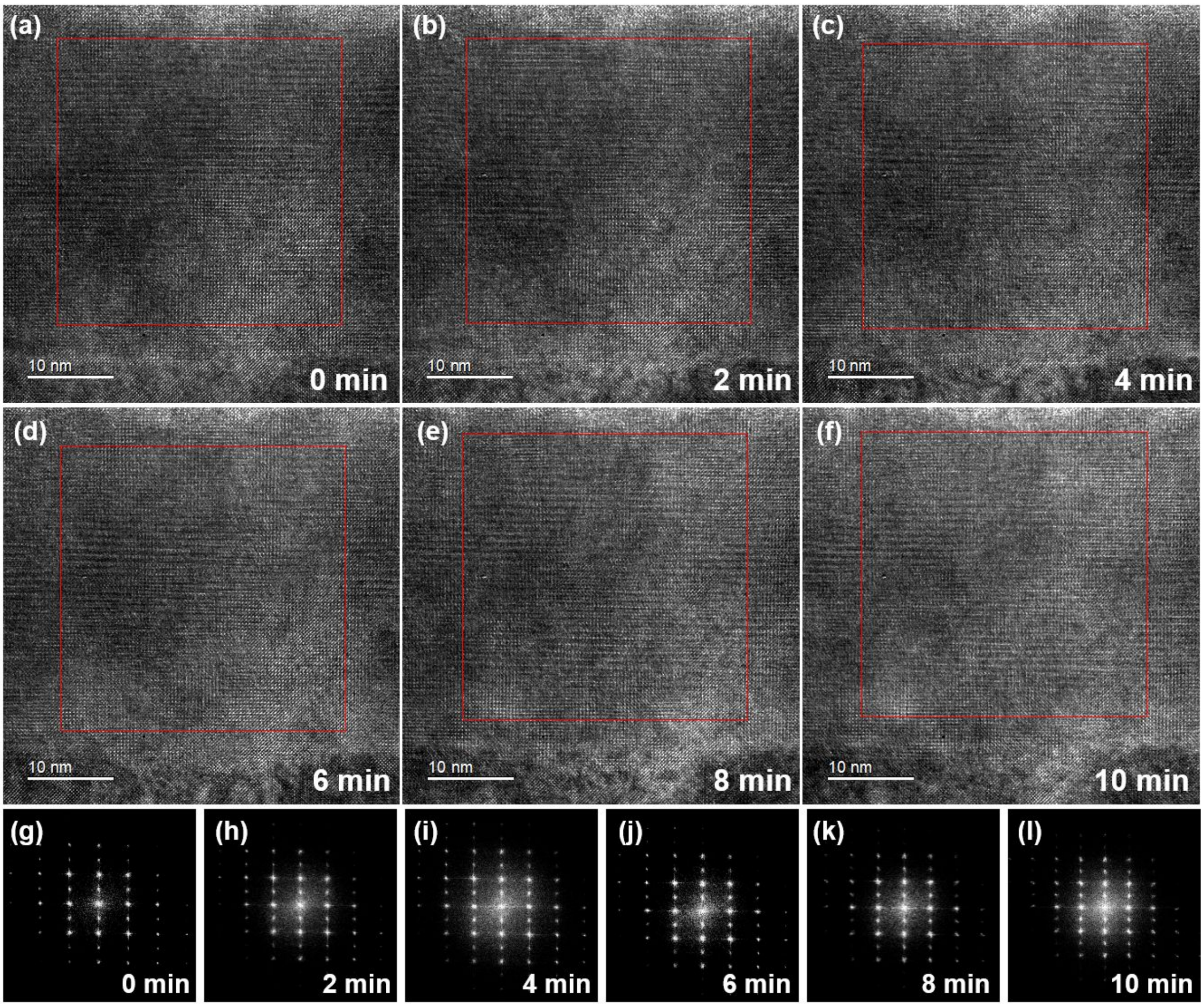

Figure S2. Electron-beam irradiation control experiment without electrical bias.

(a–f) TEM images recorded every 2 min during continuous electron-beam irradiation for 10 min under the same imaging conditions used for in situ biasing experiments. (g–l) Corresponding FFT-DPs obtained from the red-boxed regions in (a-f), respectively, exhibiting monoclinic characteristics with half-order reflections along the vertical reciprocal direction. No obvious structural transformation or disappearance of the half-order reflections is observed during beam-only irradiation.

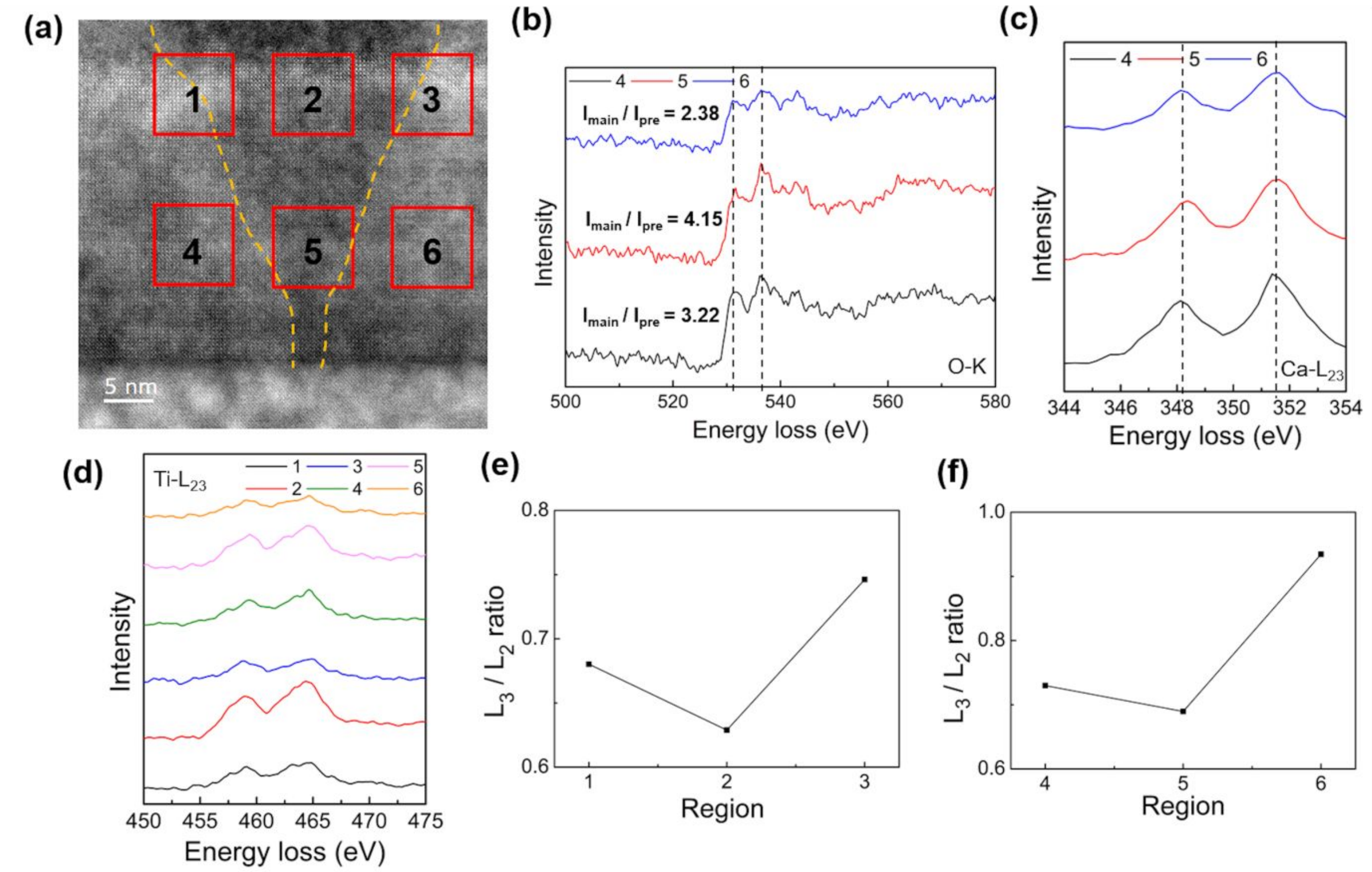

Figure S3. O K-edge, Ca L_{23}-edge, and Ti L_{23}-edge EELS spectra analysis of cycled HEO dielectric layer.

(a) ADF-STEM image of the cycled state. (b) O K-edge EELS analysis of three different regions in the cycled HEO dielectric layer. (c) Ca L_{23}-edge EELS analysis of three different regions in the cycled HEO dielectric layer. (d) Ti L_{23} edge EELS analysis of six different regions in the cycled HEO dielectric layer. (e, f) L_2/L_3 ratio analysis of the six areas.

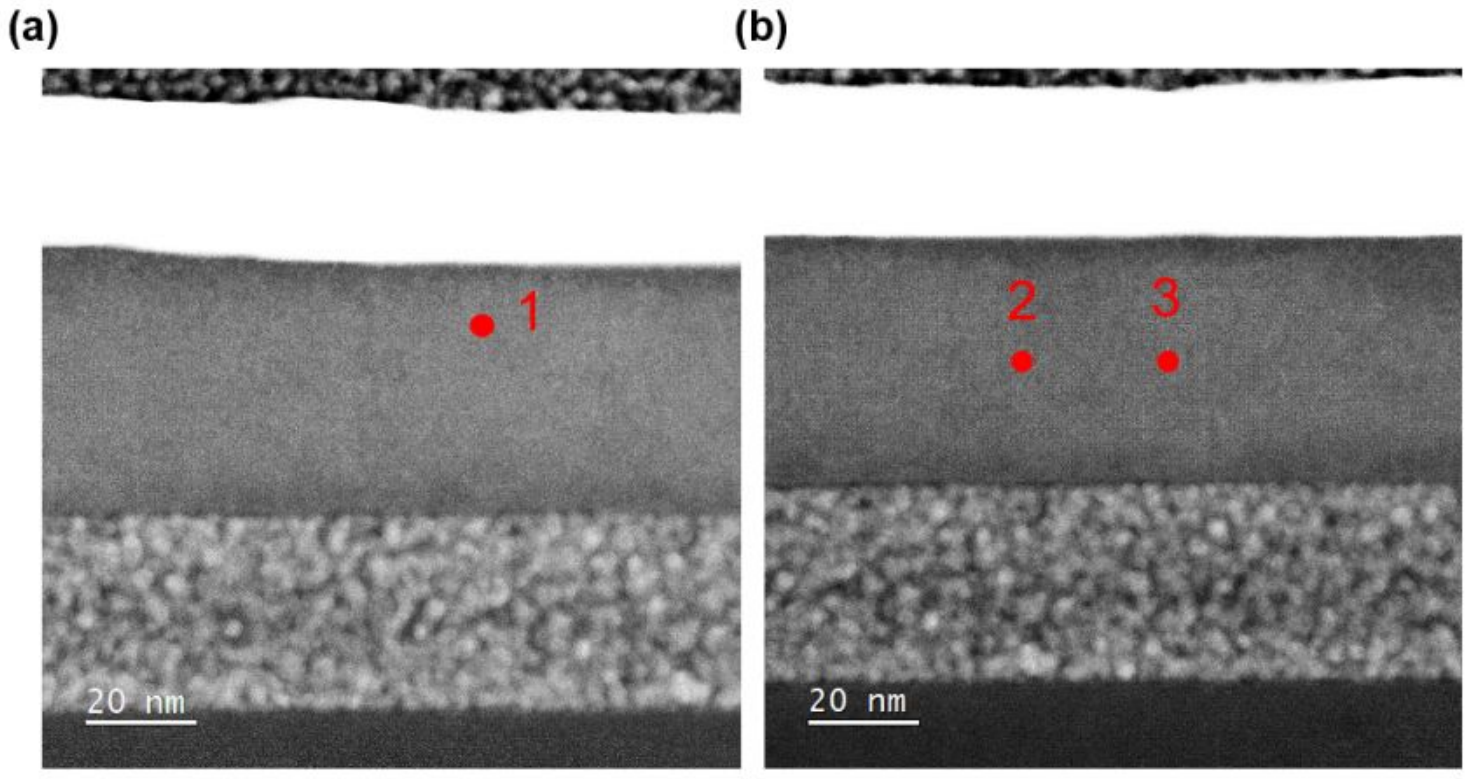

(c) EDS points results: pristine HEO layer

	Point 1	Point 2	Point 3
**O**	65.0	70.6	68.0
**Ca**	4.8	4.3	4.1
**Ti**	7.1	5.5	7.7
**Sr**	5.3	4.3	4.7
**Nb**	6.5	5.3	6.0
**Ru**	0.0	0.1	0.0
**Ta**	11.1	9.7	9.4
**Au**	0.2	0.1	0.1

Figure S4. STEM images and EDS point analyses of the pristine HEO layer.

(a,b) STEM images of the pristine HEO layer with the selected EDS acquisition points labeled. (c) Raw EDS point-analysis results obtained from the pristine HEO regions shown in (a) and (b).

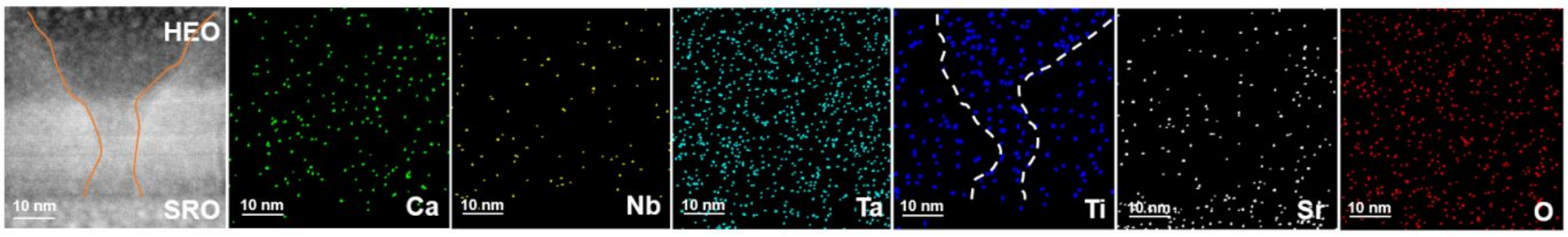

Figure S5. STEM image and EDS mapping results of Ca, Nb, Ta, Ti, Sr, and O.

The orange lines mark the transformed region identified from the STEM image.

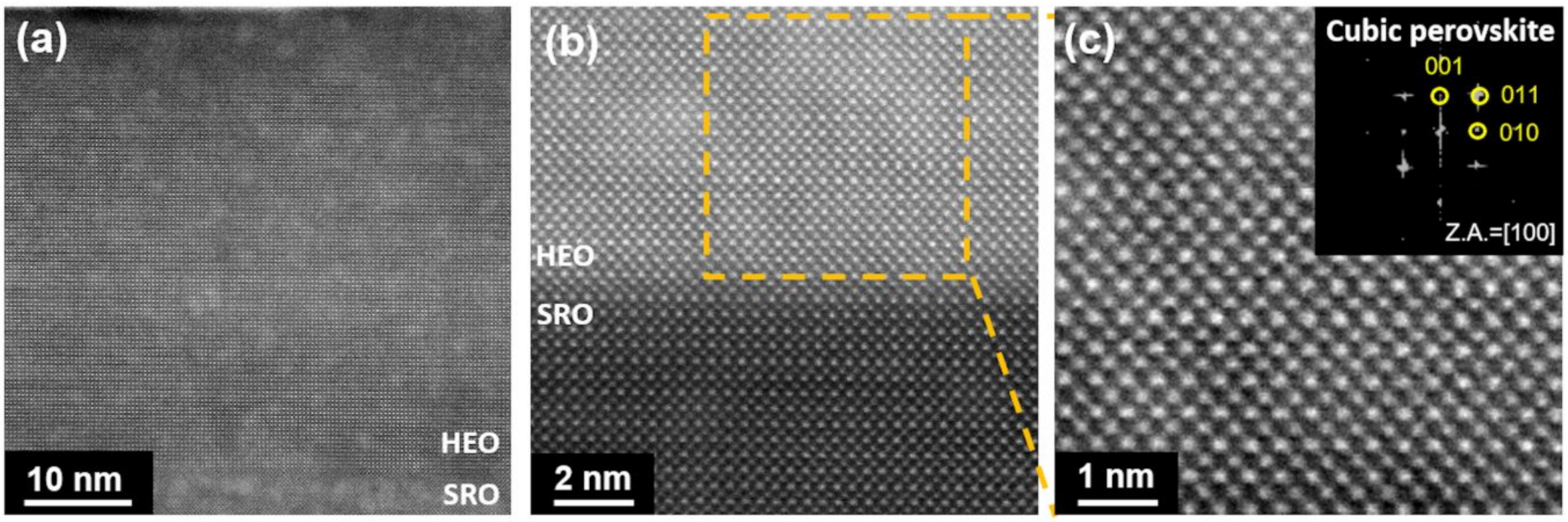

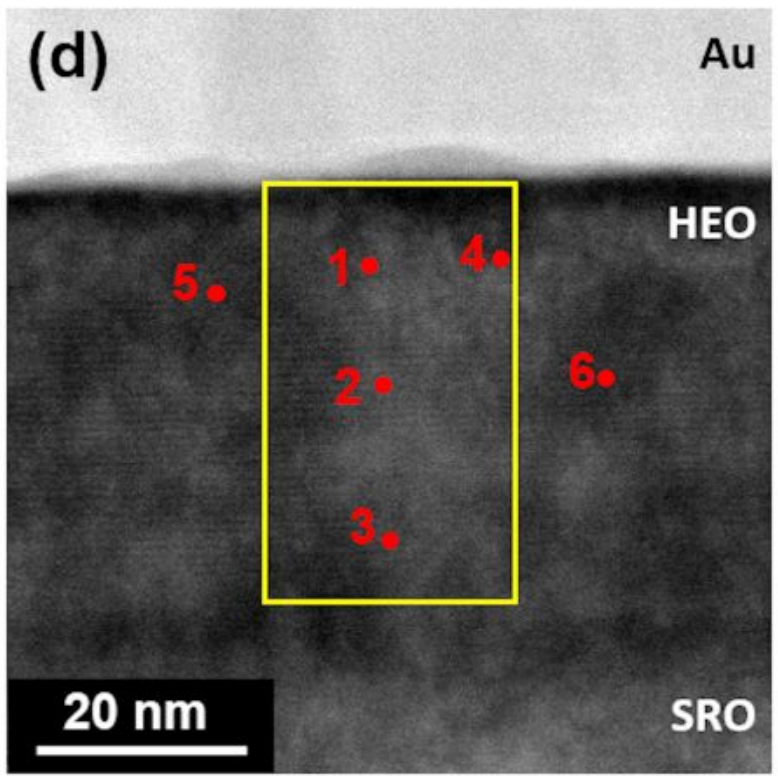

(e) EDS points results: after in situ experiment

	Point 1	Point 2	Point 3	Point 4	Point 5	Point 6
O	52.2	51.3	51.7	53.0	50.9	50.2
Ca	5.0	5.1	5.7	5.4	6.8	7.7
Ti	13.8	12.8	11.9	11.1	7.5	7.7
Sr	5.6	7.5	8.1	7.3	7.9	8.6
Nb	9.2	8.3	8.0	8.1	11.9	9.7
Ru	0.2	0.0	0.1	0.0	0.0	0.0
Ta	13.4	14.6	14.1	14.2	14.3	15.7
Au	0.6	0.4	0.2	0.9	0.8	0.3

Figure S6. STEM images and EDS points analysis of the HEO layer after the *in situ* experiment.

(a, b) STEM image after the in situ experiment. (c) Enlarged STEM image and FFT-DP for HEO within the orange-dashed region in (b). (d) STEM image of the HEO layer after the in situ experiment, with marked EDS acquisition points. (e) EDS point results of (d).

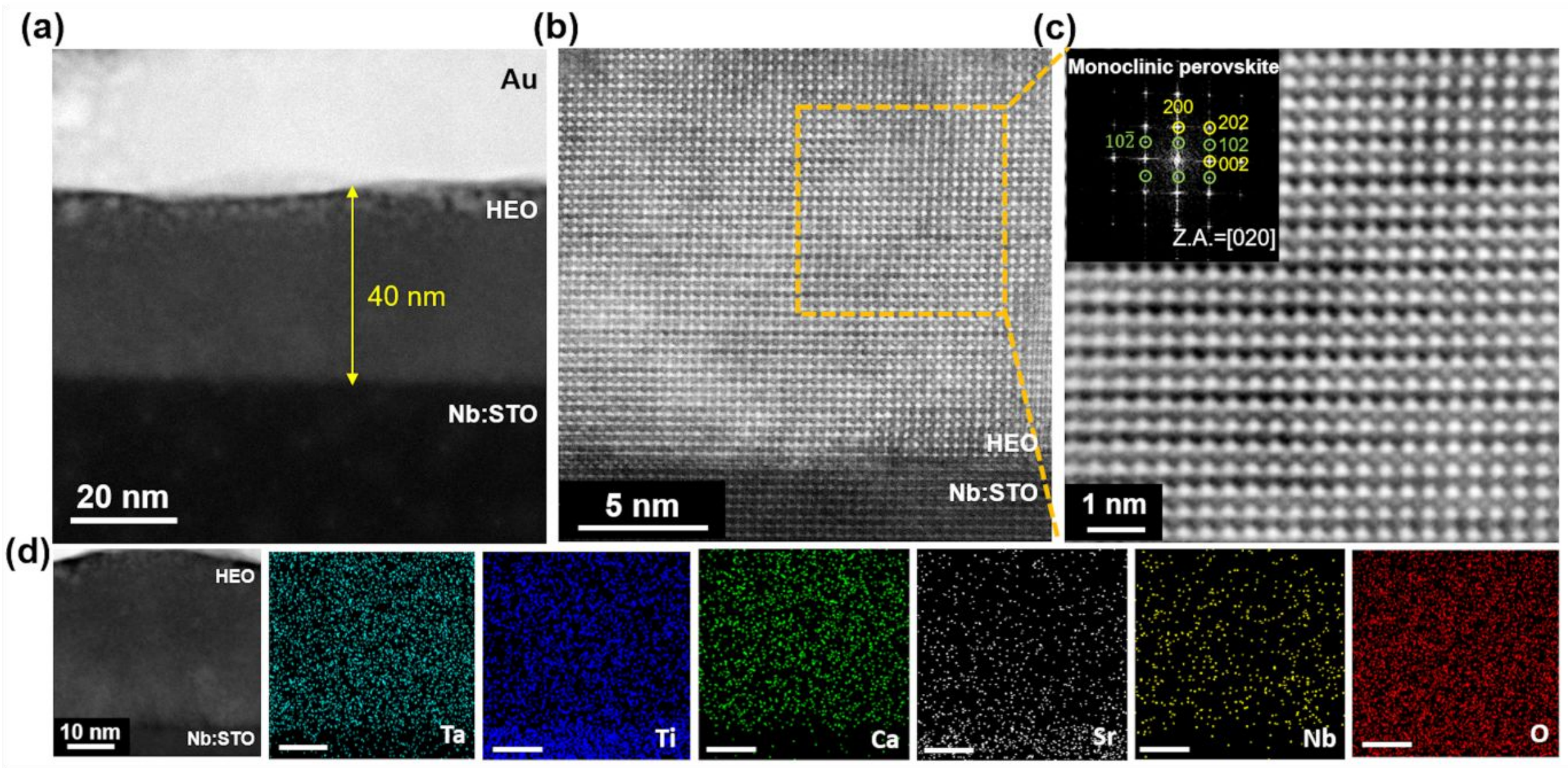

Figure S7. Basic characterizations of the pristine Au/HEO/Nb:STO device.

(a) Cross-sectional STEM image of the pristine RRAM device. (b) STEM image of the HEO layer. (c) Enlarged STEM image and FFT-DP for HEO within the orange-dashed region in (b), showing the monoclinic perovskite structure. (d) STEM image and EDS mapping results of Ta, Ti, Ca, Sr, Nb, and O.

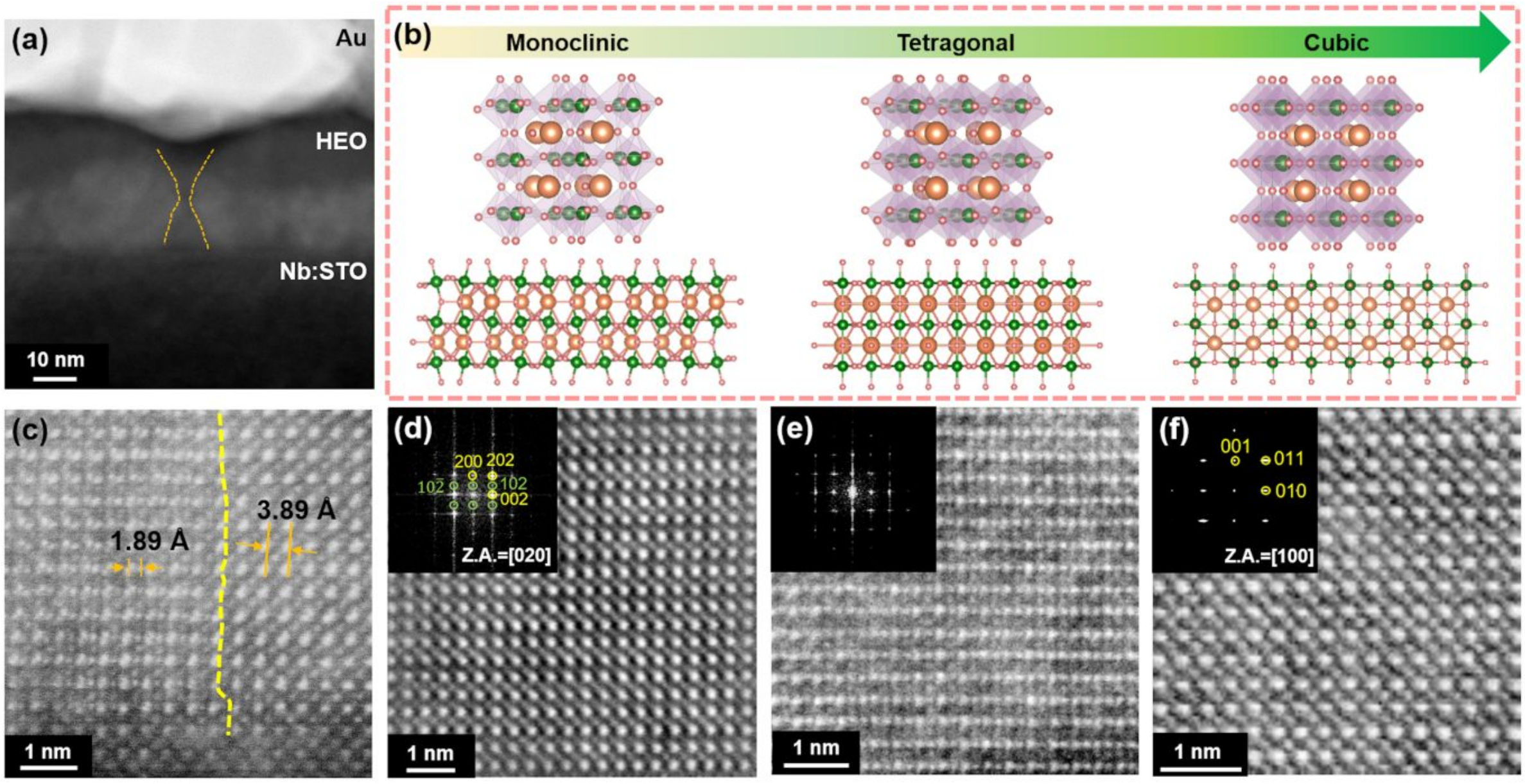

Figure S8. Illustration and STEM images of the structural transformation of perovskite-HEO.

(a) STEM image of Au/HEO/Nb:STO device after cycled electrical measurements. (b) Schematic of the monoclinic, tetragonal, and cubic perovskite, where orange spheres represent A-site atoms, and green and pink represent B-site atoms and oxygen. (c) STEM image of the cycled HEO layer of Au/HEO/Nb:STO device, demonstrating the coexistence of tetragonal and cubic perovskite structures. (d–f) STEM images and FFT-DPs of monoclinic, tetragonal, and cubic perovskite structures, indicating structural transformation with decreasing degrees of lattice distortion.

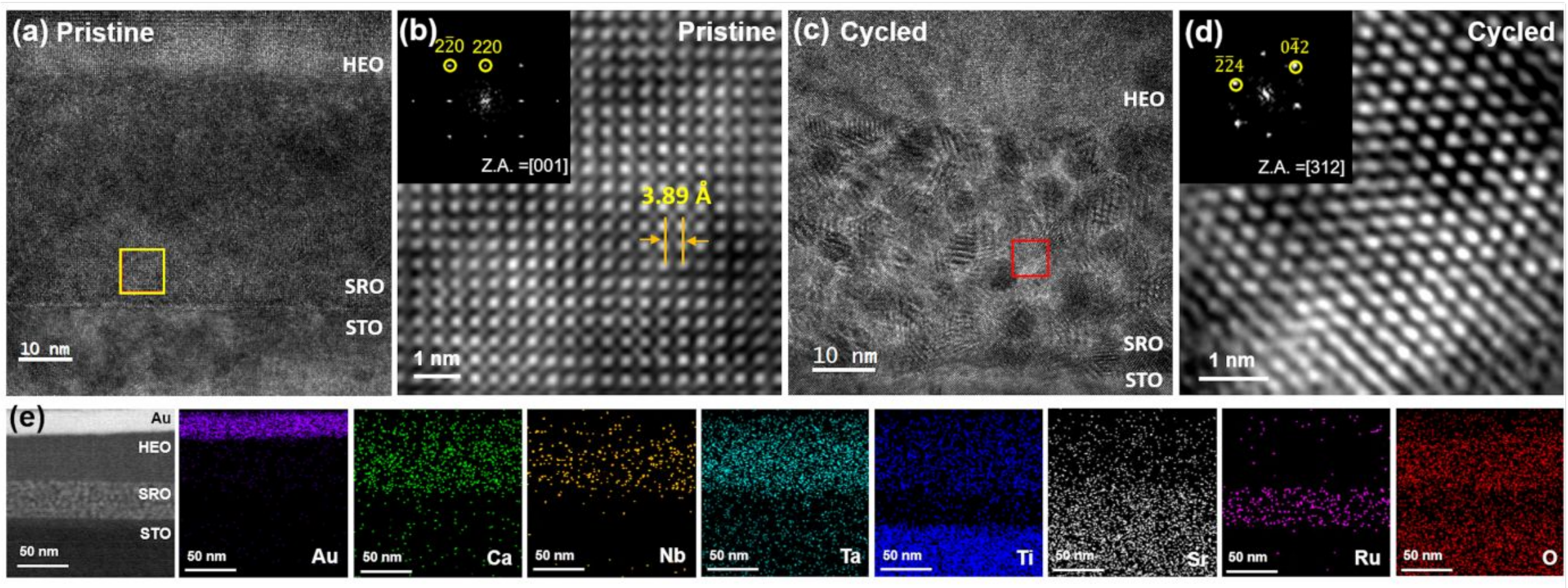

Figure S9. TEM images and EDS maps of the initial and cycled SRO layer.

(a) Cross-sectional TEM image of pristine SRO layer. (b) Enlarged HRTEM image and FFT-DP for SRO within the orange-dashed region in (a), with [001] zone axis. (c) Cross-sectional TEM image of the cycled SRO layer, indicating the local distortion of the SRO layer. (d) Enlarged HRTEM image and FFT-DP for SRO within the red-dashed region in (c), with [312] zone axis. (e) STEM image and EDS mapping results of Au, Ca, Nb, Ta, Ti, Sr, Ru, and O.

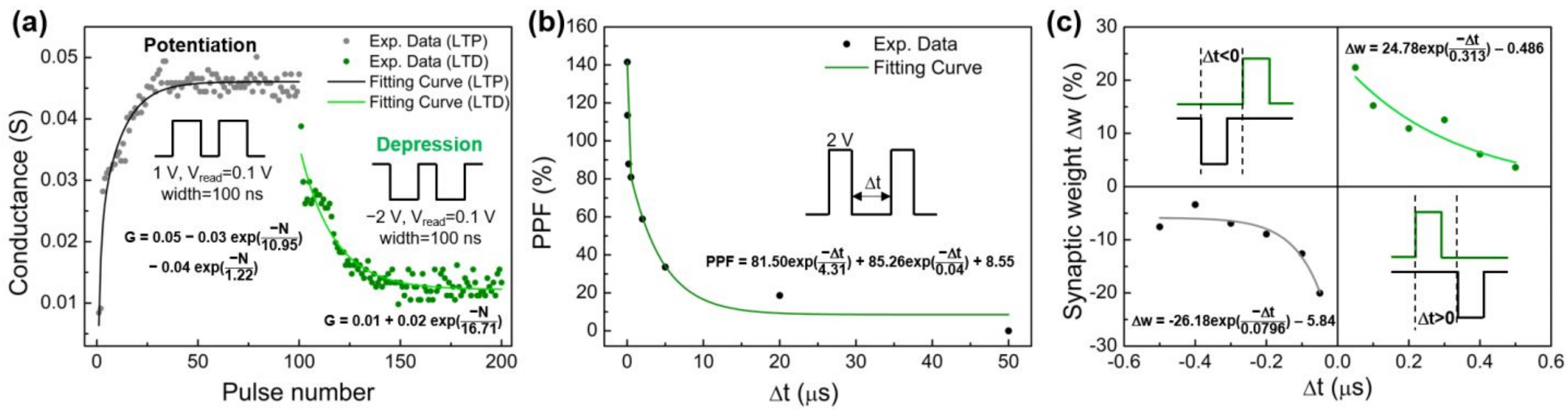

Figure S10. Synaptic plasticity characteristics of HEO-based RRAM devices.

(a) LTP and LTD were measured under 100 positive and negative pulses. (b) Δt-dependent PPF behavior demonstrating short-term synaptic plasticity with an exponential decay fitting curve. (c) Spike-timing-dependent plasticity behavior showing the dependence of synaptic weight change (Δw) on the timing difference (Δt) between pre- and post-spikes. Δw is defined as $\Delta w = (G_{after} - G_{before})/G_{before}$, where G_{before} and G_{after} are the conductances before and after the application of paired spikes, respectively.

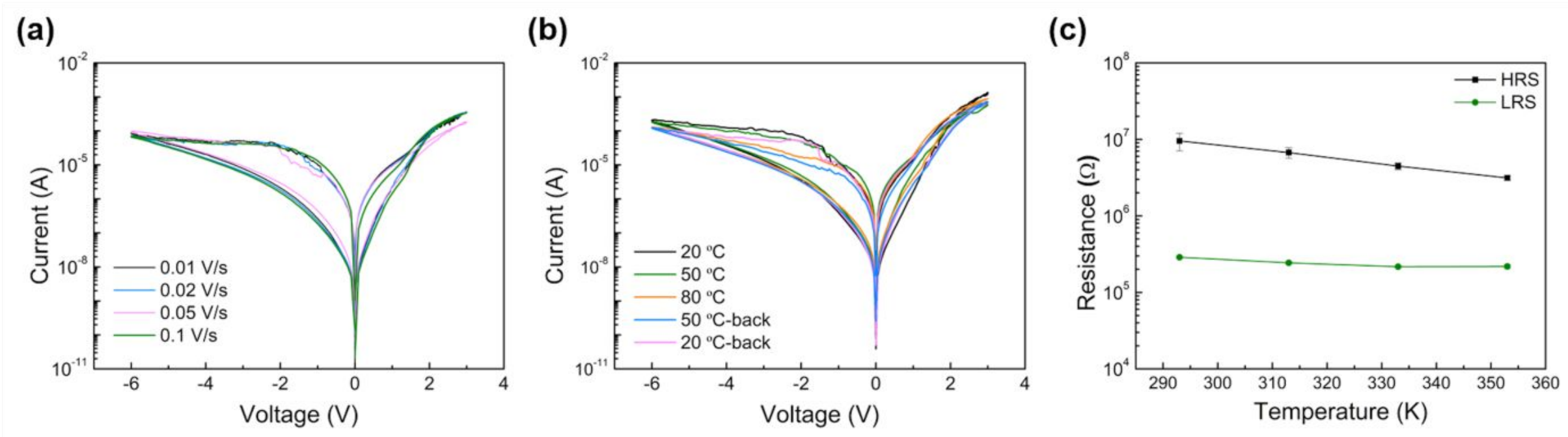

Figure S11. Electrical properties of the Au/HEO/Nb:STO device at varying sweep rates and temperatures.

(a) Comparison of *I*–*V* curves at various sweep rates (0.01, 0.02, 0.05, and 0.1 V/s). The results indicate a slightly reduced on/off ratio at a faster sweep rate, 0.1 V/s. (b) *I*–*V* curves under various temperatures (20 ℃ to 80 ℃, then back to 20 ℃). (c) Temperature-resistance characteristics of HRS and LRS at the reading voltage of −0.1 V.

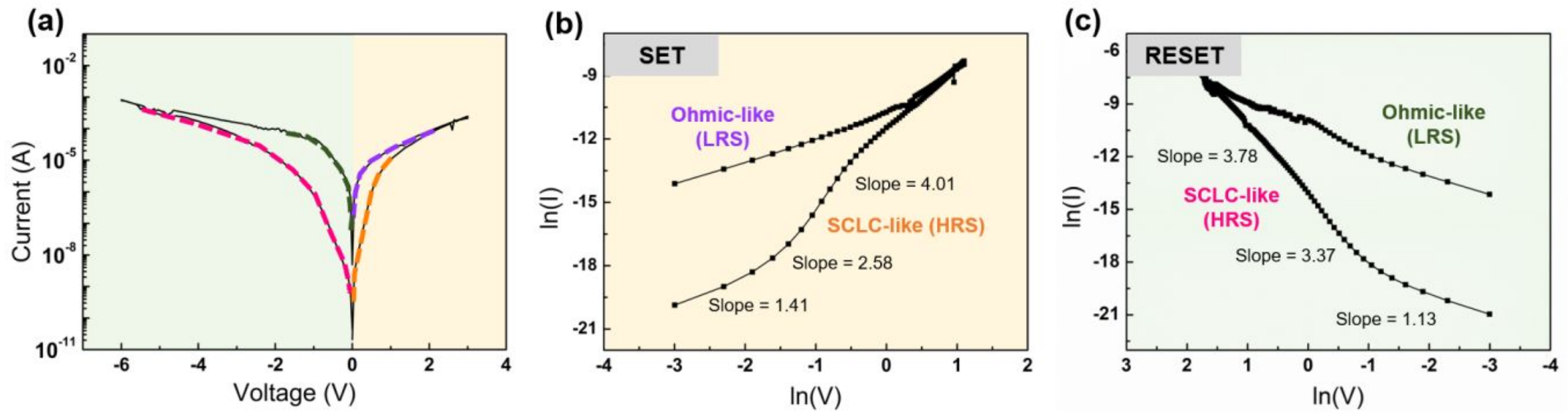

Figure S12. Electrical measurements of the transport behavior of the device.

(a) I–V curve for Au/HEO/Nb:STO device. The highlighted regions indicate the voltage ranges used for ln(I)–ln(V) analysis. (b) ln(I)–ln(V) plot during the (b) SET and (c) RESET processes. The HRS exhibits strongly nonlinear behavior with slopes larger than 2, suggesting trap-controlled SCLC-like transport, whereas the LRS shows near-Ohmic behavior.

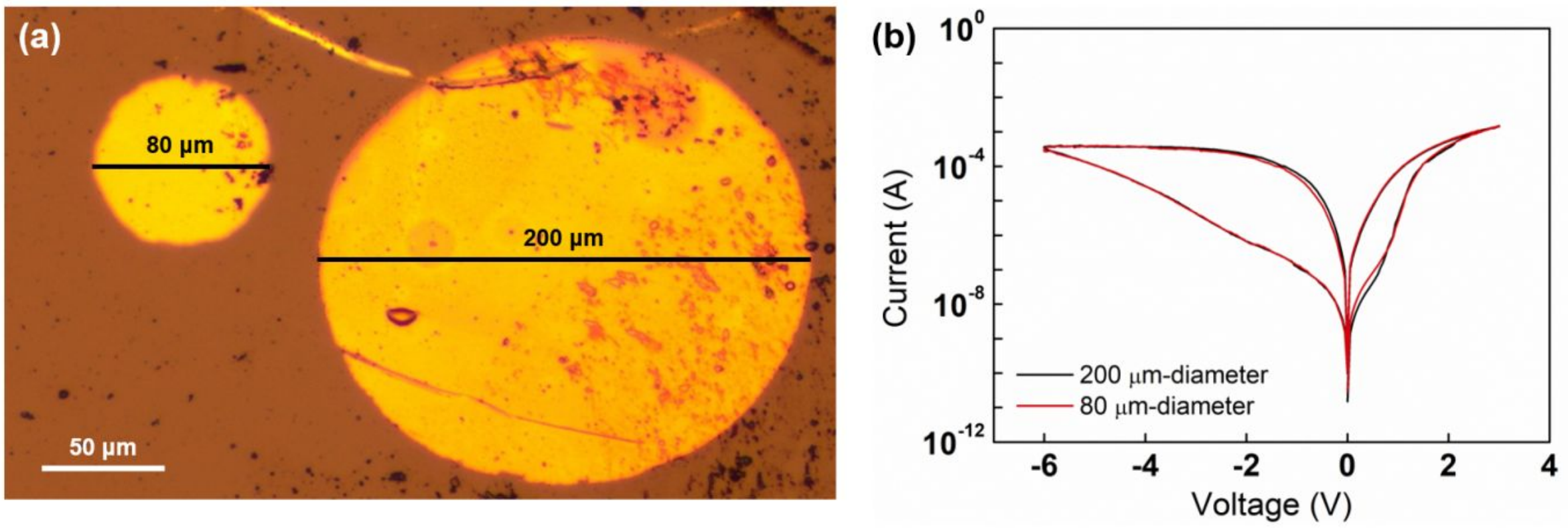

Figure S13. Electrical properties of Au/HEO/Nb:STO device at different electrode sizes. (a) Optical microscopy image of Au top electrodes with diameters of 80 and 200 µm deposited on the HEO film. (b) *I–V* curves measured from devise with 80 and 200 µm-diameter top electrodes.

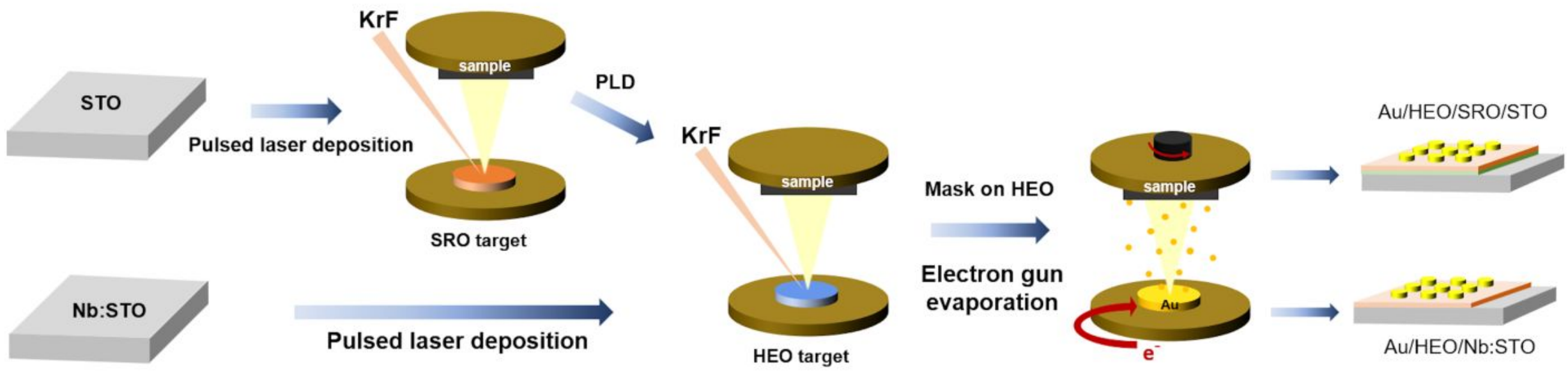

Figure S14. Fabrication process of Au/HEO/SRO/STO and Au/HEO/Nb:STO RRAM devices.

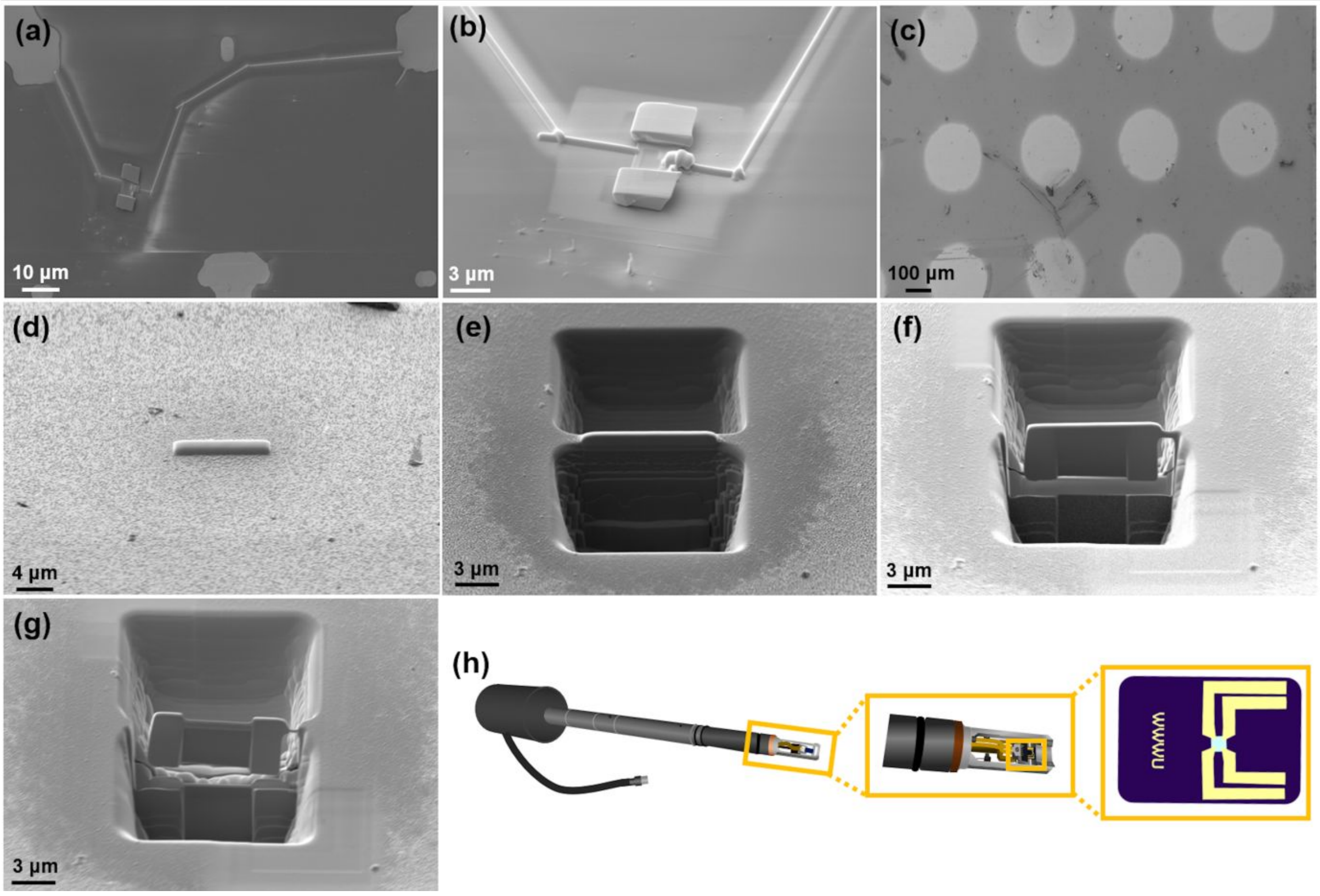

Figure S15. In situ TEM lamella specimens prepared using the focus ion beam system.

(a, b) SEM image after depositing wires on the in situ specimen. (c) SEM image of the top view of devices. (d–g) FIB experimental process of the in situ TEM specimen. (h) Illustration of the electrifying in situ holder and chip.
